# Interactional similarities and differences in the protein complex of PCNA and DNA replication factor C between rice and *Arabidopsis*

**DOI:** 10.1186/s12870-019-1874-z

**Published:** 2019-06-14

**Authors:** Jie Qian, Yueyue Chen, Yaxing Xu, Xiufeng Zhang, Zhuang Kang, Jinxia Jiao, Jie Zhao

**Affiliations:** State Key Laboratory of Hybrid Rice, College of Life Sciences, Wuhan University, Wuhan, China

**Keywords:** *Arabidopsis thaliana*, *Oryza sativa*, Replication factor C, Proliferating cell nuclear antigen, Protein-protein interaction

## Abstract

**Background:**

Proliferating cell nuclear antigen (PCNA), a conserved trimeric ring complex, is loaded onto replication fork through a hetero-pentameric AAA+ ATPase complex termed replication factor C (RFC) to maintain genome stability. Although architectures of PCNA-RFC complex in yeast have been revealed, the functions of PCNA and protein-protein interactions of PCNA-RFC complex in higher plants are not very clear. Here, essential regions mediating interactions between PCNA and RFC subunits in *Arabidopsis* and rice were investigated via yeast-two-hybrid method and bimolecular fluorescence complementation techniques.

**Results:**

We observed that OsPCNA could interact with all OsRFC subunits, while protein-protein interactions only exist between *Arabidopsis* RFC2/3/4/5 and AtPCNA1/2. The truncated analyses indicated that the C-terminal of *Arabidopsis* RFC2/3/4/5 and rice RFC1/2 is essential for binding PCNA while the region of rice RFC3/4/5 mediating interaction with PCNA distributed both at the N- and C-terminal. On the other hand, we found that the C- and N-terminal of *Arabidopsis* and rice PCNA contribute equally to PCNA-PCNA interaction, and the interdomain connecting loop (IDCL) domain and C-terminal of PCNAs are indispensable for interacting RFC subunits.

**Conclusions:**

These results indicated that *Arabidopsis* and rice PCNAs are highly conserved in sequence, structure and pattern of interacting with other PCNA monomer. Nevertheless, there are also significant differences between the *Arabidopsis* and rice RFC subunits in binding PCNA. Taken together, our results could be helpful for revealing the biological functions of plant RFC-PCNA complex.

**Electronic supplementary material:**

The online version of this article (10.1186/s12870-019-1874-z) contains supplementary material, which is available to authorized users.

## Background

Faithful transmission of accurate genome to progenies is vitally important for all living species. To achieve this, cells carried out highly processive, error-free replication of the genome in S-phase, and efficient repair of any DNA damage or misincorporated nucleotides. During these processes, a sliding clamp and its corresponding clamp loader are indispensable [1]. The ring-shaped clamp, which can encircle and slide freely along DNA, was originally studied for its role in stimulating DNA polymerases [2]. In eukaryotes, the clamp is a homotrimer termed proliferating cell nuclear antigen (PCNA) whose monomer consists of two domains [3, 4]. Previous studies have showed that all known PCNAs from different species are conserved in amino acid sequences, structures, and functions [5].

In yeast and human, PCNA is loaded onto the primer-template junction by RFC complex to tether the DNA polymerase and assure the high-speed duplication when DNA replication begins [6]. RFC is a hetero-pentameric complex whose members all belong to the AAA+ family of ATPase. Each RFC subunit (RFC1 through RFC5) consists of three domains, a N-terminal P-loop ATPase domain for binding ATP, a small α helical domain, and a five-helix bundle C-terminal domain that oligomerizes with the C-terminal of other RFC subunits to form a collar-like structure that holds the complex together as a circular-shaped hetero-pentamer [7]. A crystal structure of the yeast RFC-PCNA complex revealed that the C-terminal end of the clamp-interacting helix and the loop following it in RFC1, RFC3, and RFC4 mediated the interactions between RFC and PCNA [8]. Each of the five eukaryotic RFC subunits except RFC5 has a functional ATP-binding site and three of these ATP-binding sites are needed for loading PCNA, the site of RFC1 is not essential for clamp loading [9]. Once opened, PCNA ring clamp must be positioned by RFC complex on the DNA specifically at the primer-template junction where the polymerase is to be recruited. Results from in vitro experiment indicated that RFC has a powerful ability to rapidly scan single- and double-stranded DNA and form a stable complex with primer-template DNA although it also has high affinity for single- and double-stranded DNA [10].

Once RFC recognizes and binds a primed-DNA site, ATPase activity of the RFC subunits are activated and the ordinal ATP hydrolysis (RFC2 → RFC3 → RFC4 → RFC1) leads to closure of PCNA clamp and ejection of the RFC complex from PCNA and DNA, leaving PCNA loaded onto DNA [11, 12]. Stabilization of the PCNA clamp in an open state requires ATP binding to RFC, but not ATP hydrolysis. ATP binding to RFC3 initiates RFC activation and the clamp loader adopts a spiral conformation that stabilizes PCNA in a corresponding open spiral, and RFC2 activity contributes the most to rapid primer-template DNA release [13]. The function of RFC complex is so fundamental that disruption of any RFC subunit leads to S-phase arrest of the cell cycle in yeast [14]. Mutation of *Drosophila RFC4* causes striking defects in DNA replication and checkpoint control [15]. In *Aspergillus nidulans*, mutation of *AnRFC1* leads to increased mitotic recombination and mutation, suggesting that *AnRFC1* is essential for DNA replication and UV repair [16]. In *Arabidopsis*, *RFC1* plays an essential role in mediating genome stability and transcriptional gene silencing [17]. Other studies have shown that *AtRFC1* also participates in meiotic homologous recombination [18, 19]. *AtRFC3* is involved in negative regulation of systemic acquired resistance, and *AtRFC4* is critical for DNA replication during the mitotic cell cycle [20, 21].

Since DNA synthesis on the lagging strand is discontinuous and primers are being synthesized every 100–200 nt to generate Okazaki fragments, PCNA is required to be loaded at each Okazaki fragment and accumulates on the lagging strand [22]. In addition to its key role in DNA replication, PCNA also acts as a platform for recruiting participators of the DNA damage response and checkpoint machineries [23]. Following, release of the RFC complex from replication forks allows DNA polymerases to bind PCNA and initiate DNA synthesis. On the other hand, PCNA participates in regulating protein degradation of its binding partners during replication. For example, the replication licensing factors, cell division control protein 6 (CDC6), and chromatin licensing and DNA replication factor 1 (CDT1) are degraded to prevent re-replication of DNA when they were bound to PCNA on chromatin and modified by the Cullin 4-DDB1-CDT2 (CRL4^CDT2^) E3 ubiquitin ligase during S-phase [24].

During a single replication cycle, PCNA interacts with numerous proteins involved in normal DNA replication, chromatin assembly, DNA damage repair, and checkpoint response. The inner surface of each PCNA monomer is formed by twelve positively charged α-helices that interact with DNA, and the outer layer contains fifty-four β-sheets and one IDCL domain for protein-protein interactions [25, 26]. A general motif governing PCNA-protein interactions is the PCNA-interacting protein (PIP) box, a short sequence motif that is present in RFC1 and RFC3, and most other PCNA-binding proteins [8, 27, 28]. It has been demonstrated that PCNA and its post-translationally unmodified form can directly interact with over 200 proteins that are involved in DNA replication (polymerase-δ), DNA repair (polymerase-τ, polymerase-κ, and polymerase-η), cell cycle regulatory proteins (p21, p53, and p35), chromatin accessibility (HDAC1), and transcription (p65) [25, 29, 30]. However, the molecular mechanism of how PCNA bind to different partners with different affinities is not so clear.

In *Arabidopsis*, AtPCNA1 and AtPCNA2 share such a high identity (97%) that there are only nine different amino acid residues. Although great achievements have been accomplished on biological functions of the eukaryotic PCNA over the last decades, the functional relevance of AtPCNA1/2 and how they are loaded is still unclear in higher plants. Crystal structure analysis showed no obvious difference between AtPCNA1 and AtPCNA2 ring clamps, and they can form another two kinds of heterotrimers (PCNA1-PCNA1-PCNA2 or PCNA1-PCNA2-PCNA2) in vitro [31, 32]. It has also reported that AtPCNA2, but not AtPCNA1, could functionally interact with the *Arabidopsis* translesion DNA polymerase η and λ, implying that AtPCNA1 and AtPCNA2 may have functional differences in DNA repair [33, 34].

Although great progress has been made in illustrating the three-dimensional structures and biological functions of PCNA clamps in yeast and human, little is known about the composition of PCNA and its binding partners in higher plants. Via a yeast system, it has reported that both AtPCNA1 and AtPCNA2 were able to functionally take the place of the essential roles of yeast PCNA [35], implying that there might be functional redundancies between the two *Arabidopsis* PCNAs. On the other hand, direct interactions between AtPCNA1 and AtPCNA2 had been proved. The two PCNAs possibly form homo- and hetero-trimeric complexes, and may play critical roles in cellular signal transduction [31, 32]. So, it is of great significance to reveal the biological functions of plant PCNA via investigating the interaction relationship between PCNA and RFC complex and analyzing the possible way of RFCs loading PCNAs.

In this study, sequence homology of rice (*Oryza sativa*) and *Arabidopsis* PCNAs were analyzed. Via employing yeast-two-hybrid (Y2H) method and bimolecular fluorescence complementation (BiFC) techniques, we investigated the interactions between PCNA and RFC subunits. Meanwhile, a series of truncated proteins were used to identify the essential interacting regions between them. Our studies would provide new ideas to further reveal the biological functions of PCNA in higher plants.

## Results

### AtPCNA1/2 and OsPCNA are highly conserved and widely expressed in different tissues

To investigate the conservation of PCNA proteins, we performed full-length alignment of the amino acid sequences of PCNAs in *Arabidopsis*, rice, human, mouse, yeast, and so on. The results showed that PCNAs exhibit high identity in amino acid sequences and contain a conserved lysine-164 (Additional file 1a, asterisk), which has been proved to be essential for responding to DNA damage or stalled replication forks [25]. The IDCL domain was also found in AtPCNA1/2 and OsPCNA. To further study the conservation of sliding clamps in different species, phylogenetic analysis of PCNAs from various species was performed, revealing that PCNAs exist widely in the most of eukaryotes (Additional file 1b). All these results indicated that AtPCNA1/2 and OsPCNA are highly conserved and share great similarity.

To characterize the expression patterns of the *AtPCNA1/2 and OsPCNA* genes, quantitative real-time PCR (qRT-PCR) was performed to evaluate their relative transcript levels in various tissues, respectively. The results showed that *AtPCNA1 and AtPCNA2* genes share similar expression patterns in almost all vegetative and reproductive tissues, especially in inflorescences (Additional file 2a-b). Similarly, the transcript of *OsPCNA* gene is detected in almost all vegetative and reproductive tissues with the highest expression in leaves and inflorescences (Additional file 2c).

### Stable interactions were observed between RFC subunits and PCNAs in rice and *Arabidopsis*

**Fig. 1:**
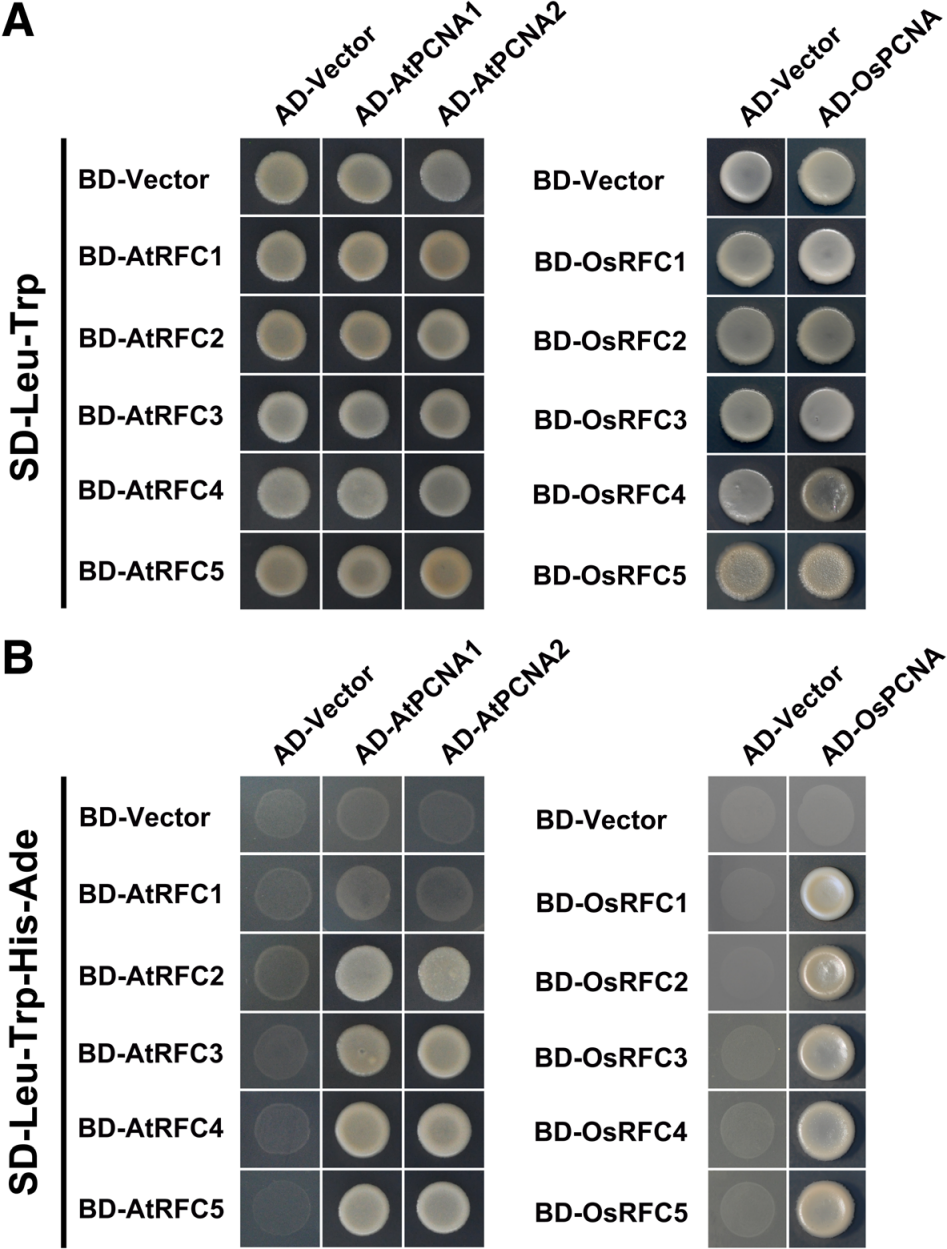
Yeast-two-hybrid assay to assess physical interactions between PCNA and RFC subunits of *Arabidopsis* and rice. The co-transformed strains are spotted on SD-Leu-Trp (**a**) and SD-Leu-Trp-His-Ade (**b**) plates to test the physical interactions between the candidate proteins. Yeast strains co-transformed with the ‘empty’ AD or BD plasmids are used as negative controls. AD, *pGADT7* vector; BK, *pGBKT7* vector; SD, synthetic dextrose

**Fig. 2:**
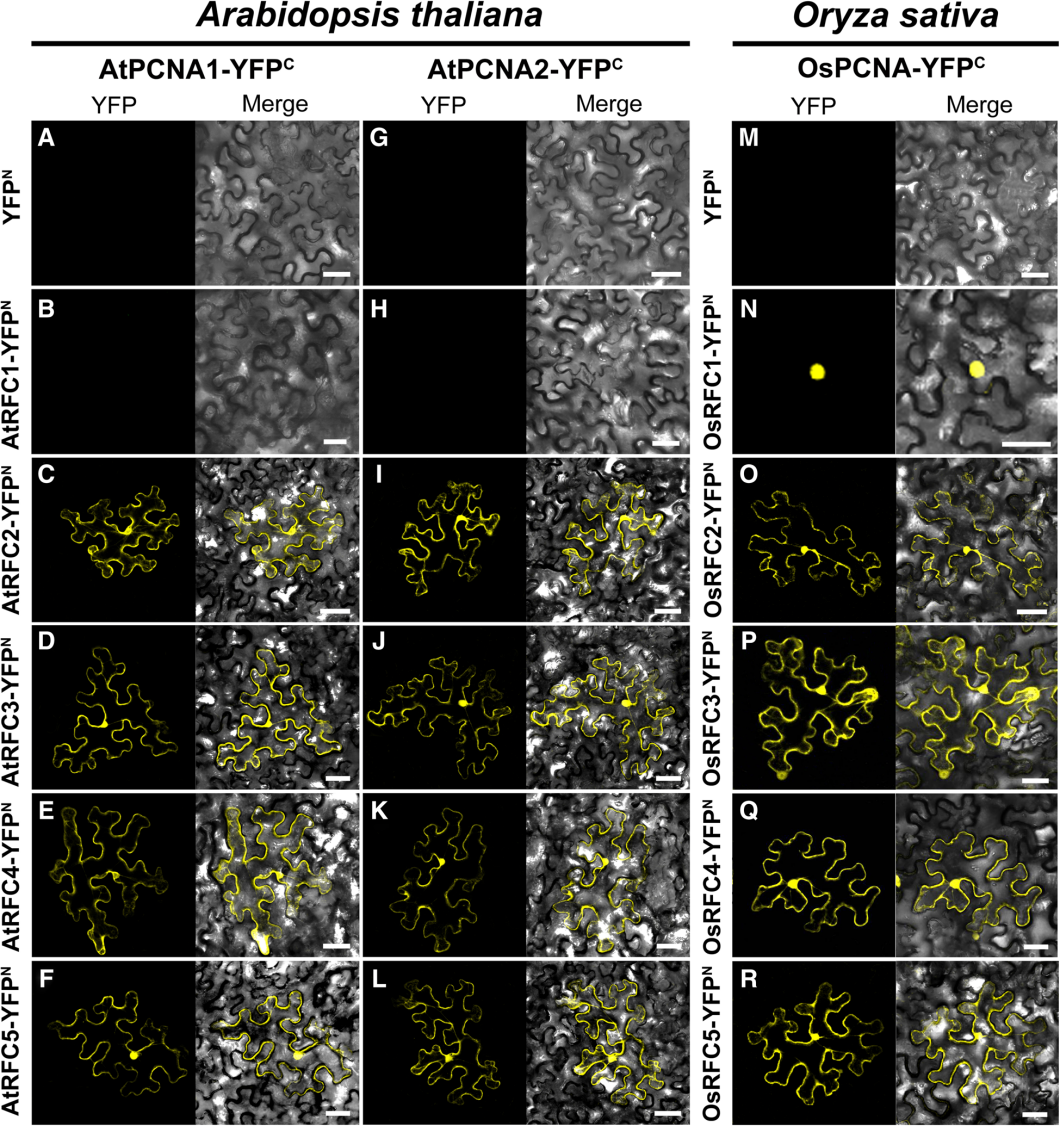
BiFC assays in epidermis cells of tobacco leaf to identify the interactions between PCNA and RFC subunits of *Arabidopsis* and rice. **a** and **b** No YFP signal is detected between AtPCNA1-YFP^C^ and YFP^N^/AtRFC1-YFP^N^. **c**-**f** AtPCNA1 can interact with AtRFC2/3/4/5, respectively. g and **h** No YFP signal is detected between AtPCNA2-YFP^C^ and YFP^N^/AtRFC1-YFP^N^. **i**-**l** AtPCNA2 can interact with AtRFC2/3/4/5, respectively. **m** No YFP signal is detected between OsPCNA-YFP^C^ and YFP^N^. **n**-**r** OsPCNA can directly interact with OsRFC1/2/3/4/5. The tobacco epidermal cells are co-transfected with constructs encoding the candidate fusion proteins. YFP^C^, the C-terminal fragment of YFP (156–239 aa); YFP^N^, the N-terminal fragment of YFP (1–155 aa). Bars = 50 μm

It has been proved in yeast and human that direct interactions exist between RFC subunits and PCNA clamp [8, 36, 37]. To investigate the interacting patterns between PCNA and the RFC complex in *Arabidopsis* and rice, we employed yeast-two-hybrid method to identify which RFC subunits can bind PCNAs. Because the yeast two hybrid assays are performed in live cells, any interactions detected could potentially be stabilized by or mediated by other cellular proteins. The results showed that only yeast cells harboring AtRFC2/3/4/5-BD and AtPCNA1/2-AD could survive on SD-medium lacking Leu, Trp, His, and Ade, while visible yeast could be observed among cells co-expressed OsRFC1/2/3/4/5-BD and OsPCNA-AD (Fig. 1). Afterwards, a tobacco (*Nicotiana benthamiana*) transient transformation assay was performed to confirm the above results. Interactions between these PCNA and RFC subunits were analyzed in tobacco leaf epidermal cells via BiFC technique, as many as thirty-six different combinations of RFCs-YFP^N^ and PCNA-YFP^C^ in total were tested. The results showed that no YFP signal was detected in cells harboring YFP^N^/AtRFC1-YFP^N^ and AtPCNA1/2-YFP^C^ (Fig. 2a, b, g, h). In the case of AtRFC2/3/4/5-YFP^N^ and AtPCNA1/2-YFP^C^, obvious YFP signals were observed both in the nucleus and cytoplasm (Fig. 2c-f, i-l). Meanwhile, we noticed that stable fluorescent signals of OsRFC1-YFP^N^/OsPCNA-YFP^C^ were accumulated only in the nucleus, while the interaction signals of OsRFC2/3/4/5-YFP^N^ and OsPCNA-YFP^C^ could be detected both in the nucleus and cytoplasm (Fig. 2n-r). No YFP signals were detected between YFP^N^ and OsPCNA-YFP^C^ (Fig. 2m). These results indicated that all rice RFC subunits have the ability to interact with PCNA, but in *Arabidopsis*, only AtRFC2/3/4/5 subunits have the potential to bind to AtPCNA1/2. The lack of interaction between *Arabidopsis* RFC1 and AtPCNA1/2 suggested that there may be differences between *Arabidopsis* and rice RFC subunits in recognizing and loading PCNA.

### *Arabidopsis* PCNA1/2 and rice PCNA could substitute each other to interact with RFC subunits

**Fig. 3:**
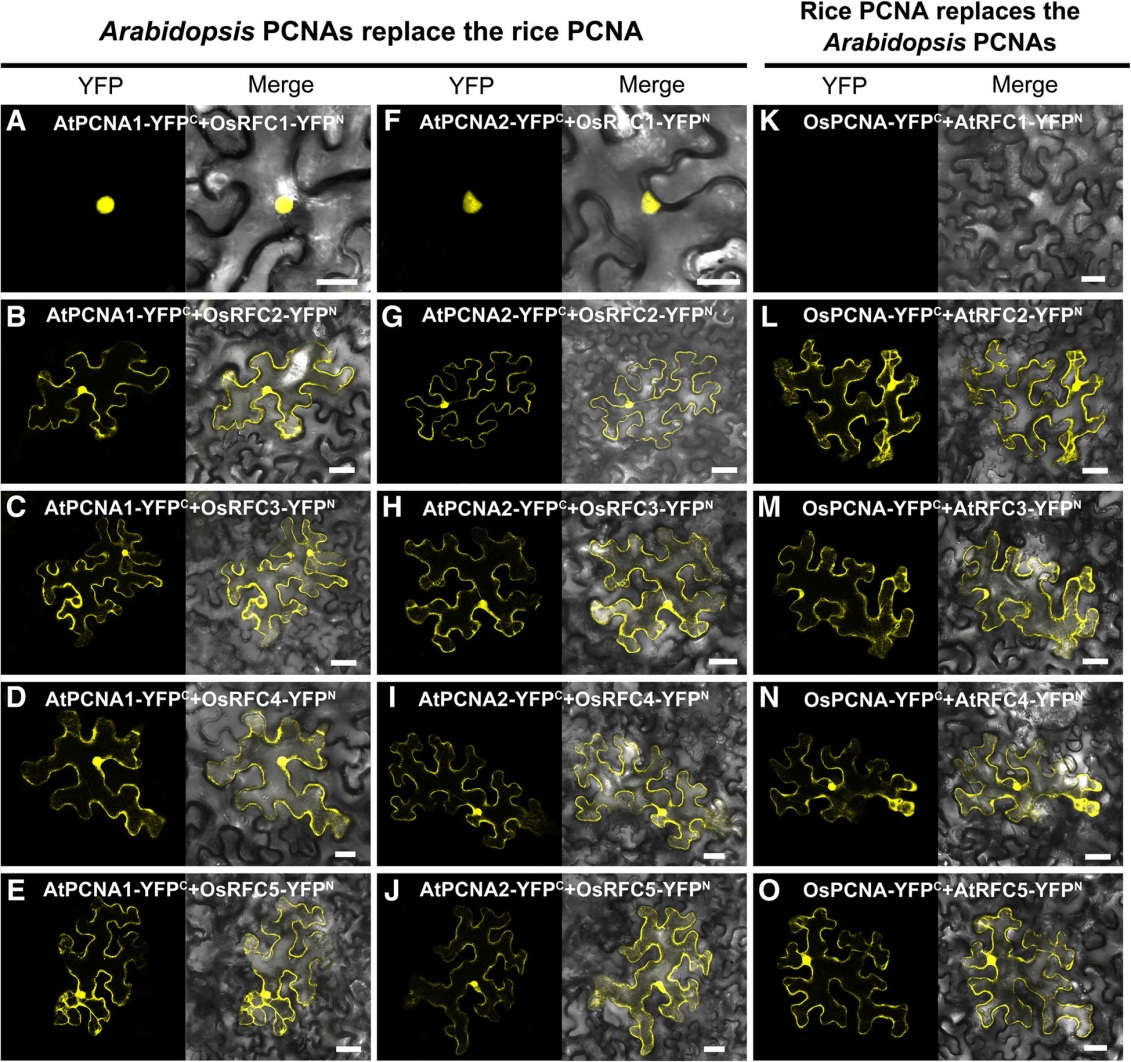
The conservative substitution of AtPCNA1/2 and OsPCNA. **a**-**j** AtPCNA1/2 can directly interact with OsRFC1/2/3/4/5. **k**-**o** OsPCNA can directly interact with AtRFC2/3/4/5. The tobacco epidermal cells are co-transfected with constructs encoding the candidate fusion proteins. YFP^C^, the C-terminal fragment of YFP (156–239 aa); YFP^N^, the N-terminal fragment of YFP (1–155 aa). Bars = 50 μm

Sequence alignment and phylogenetic analysis revealed that the PCNAs in *Arabidopsis* and rice exhibit extremely high similarities in sequence, we then test whether AtPCNA1/2 can replace OsPCNA and interact with OsRFC subunits. The results showed that stable fluorescent signals were accumulated in cells co-expressed AtPCNA1/2-YFP^C^ and OsRFC1/2/3/4/5-YFP^N^ (Fig. 3a-j). Meanwhile, interactions between OsPCNA and *Arabidopsis* RFC2/3/4/5 subunits were investigated. We observed that stable YFP signals were accumulated in tobacco epidermal cells co-expressed OsPCNA-YFP^C^ and AtRFC2/3/4/5-YFP^N^ (Fig. 3l-o), while no YFP signals in the combination of OsPCNA and AtRFC1 were detected (Fig. 3k). This indicated that the lack of interaction between *Arabidopsis* RFC1 and AtPCNA1/2 as attributed to the AtRFC1 and its partners rather than the AtPCNA1 or 2. Taken together, these results suggested that AtPCNA1/2 and OsPCNA exhibit high conservation in amino acid sequence, protein structure, and protein-protein interactions.

### Regions required for the RFC2/3/4/5 subunits to interact with PCNAs in *Arabidopsis*

**Fig. 4:**
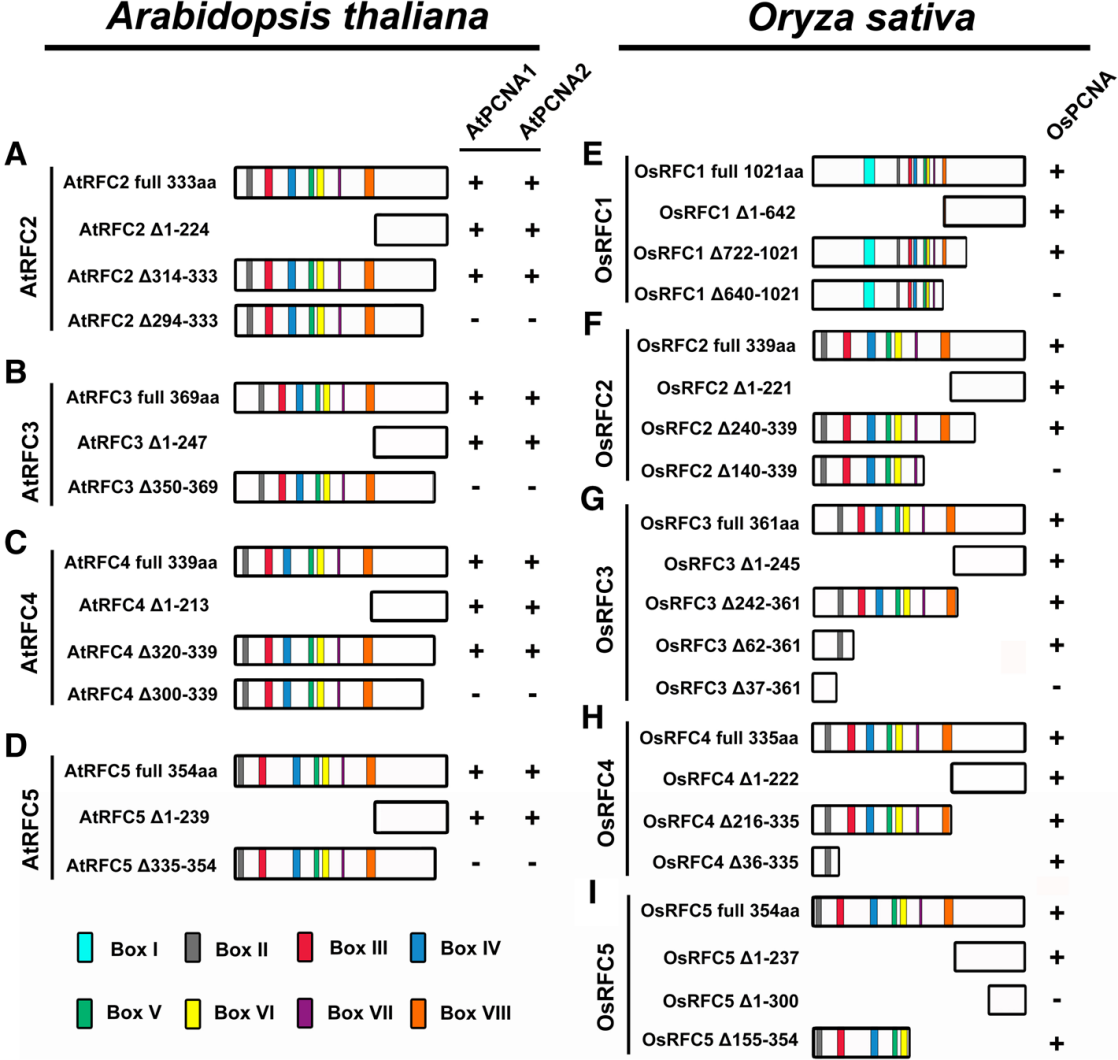
Summary of interactions between the truncated RFC subunits and PCNA in *Arabidopsis* and rice. **a**-**d** Schematic diagrams of the regions required for AtRFC complex to interact with AtPCNA1/2 in *Arabidopsis*. **e**-**i** Schematic diagrams of the regions required for RFC complex to interact with OsPCNA in rice. The symbol “+” means that direct interactions exist between these proteins, while the symbol “-” indicates that no interactions exist between these proteins; NA: not applicable

To identify essential regions of the RFC2/3/4/5 subunits for interacting with PCNA in *Arabidopsis*, a series of truncated RFC proteins were fused with N- or C-terminus of the YFP and used in the BiFC assay. As shown in Fig. 4, Additional files 3 and 4, the N-terminal 224 aa of the AtRFC2 subunit is not required for interacting with AtPCNA1/2 (Additional file 3a and b). Consistent with this, AtRFC2 Δ314–333 could still interact with AtPCNA1/2 (Additional file 3c-d). However, AtRFC2 Δ294–333, with another 20 aa of C-terminal deleted, led to no interaction with AtPCNA1/2 (Additional file 3e -f). These results indicated that the region between 294 to 313 aa of AtRFC2 mediated its interaction with AtPCNA1/2. Deletion analysis of the AtRFC3 showed that deletion of the N-terminus 1 to 247 aa did not affect the interactions between AtRFC3 and AtPCNA1/2 (Additional file 3 g-h). AtRFC3 Δ350–369, with 20 aa of C-terminal deleted, could not interact with AtPCNA1/2 (Additional file 3i-j), indicating that the C-terminal region between the 350 to 369 aa of AtRFC3 mediated its interaction with AtPCNA1/2. Similarly, AtRFC4 Δ1–213, with 213 aa of N-terminal deleted, could interact with AtPCNA1/2 (Fig. 4c, Additional file 3 k-l). AtRFC4 Δ320–339 that lacked its 20 C-terminal amino acids could also interact with AtPCNA1/2 (Additional file 3m-n), while AtRFC4 ΔC300–339, with an additional deletion of 20 C-terminal amino acids, no longer supported its connection with AtPCNA1/2 (Additional file 3o-p). These findings suggested that the C-terminal region of AtRFC4 between the 300 to 320 aa was required for the interactions with AtPCNA1/2. In the same way, the truncated AtRFC5 lacking 239 aa in its N-terminal did not affect the interactions with AtPCNA1/2 (Fig. 4d, Additional file 3q and r). However, when the 20 aa in its C-terminal was deleted (AtRFC5 Δ335–354), the above interactions disappeared (Additional file 3 s-t), demonstrating that sequences from 335 to 354 aa of the AtRFC5 C-terminal were required for the interactions with AtPCNA1/2.

### Regions required for the RFC subunits to bind PCNA in rice

To investigate the regions of OsRFC1/2/3/4/5 that were required for interacting with OsPCNA, truncated variants of RFC complex were used in the BiFC assay (Fig. 4e-i; Additional file 4). The OsRFC1 Δ1–642 that lacked 642 aa in its N-terminal did not affect the interactions with OsPCNA (Additional file 4a). Similar result was observed when deleting 300 aa of OsRFC1 in its C-terminal (Additional file 4b). However, OsRFC1 Δ640–1021, with another 82 aa deleted, could no longer interact with OsPCNA (Additional file 4c), indicating that the region between 640 to 722 aa of OsRFC1 was indispensable for binding OsPCNA. Deleting the highly conserved Boxes II-VIII of OsRFC2 (OsRFC2 Δ1–221) and a deletion of 100 aa in its C-terminal did not affect the interactions with OsPCNA (Additional file 4d-e). However, OsRFC2 Δ140–339, with another 100 aa deleted, could not bind OsPCNA (Additional file 4f). Similarly, OsRFC3 Δ1–245 that lacked 245 aa in its N-terminal and Δ242–361 and Δ62–361 that lacked 120 aa and 300aa in its C-terminal, did not affect the interactions with OsPCNA (Additional file 4 g-i). However, OsRFC3 Δ37–361, which possessed a deletion of 325 aa in its C-terminal, could not interact with OsPCNA (Additional file 4j), indicating that the regions between 37 and 62 aa of OsRFC3 were required for interacting with OsPCNA.

Next, we found that OsRFC4 Δ1–222, OsRFC4 Δ216–335 and OsRFC4 Δ36–335 still supported the interactions with OsPCNA (Fig. 4h, Additional file 4 k), indicating that the regions between 1 and 36 aa in and 222–335 aa of OsRFC4 is indispensable for interactions with OsPCNA. On the other hand, we found that removing 237 aa of N-terminal or 100 aa of its C-terminal retained the ability for binding OsPCNA (Additional file 4n and p). However, OsRFC5 Δ1–300, with 300 N-terminal amino acids deleted, lost the ability to interact with OsPCNA (Additional file 4o), suggesting that the region between 237 to 300 aa within the OsRFC5 C-terminal was indispensable for binding OsPCNA. Based on our results, we noticed that the PCNA-interacting domains of RFC subunits are quite different in *Arabidopsis* and rice. In *Arabidopsis*, the regions all located near the C-terminal of RFC2/3/4/5, while the essential domains of rice RFC subunits are closer to the N-terminal (Fig. 4). These results are not very consistent with the previous study that the N-terminal of yeast RFC subunits contribute to the interactions with PCNA clamp [8]. One possible explanation for these differences is that RFC complex and single RFC subunit may utilize different regions to bind PCNA.

### Regions required for PCNA-PCNA interactions of *Arabidopsis* and rice PCNA

**Fig. 5:**
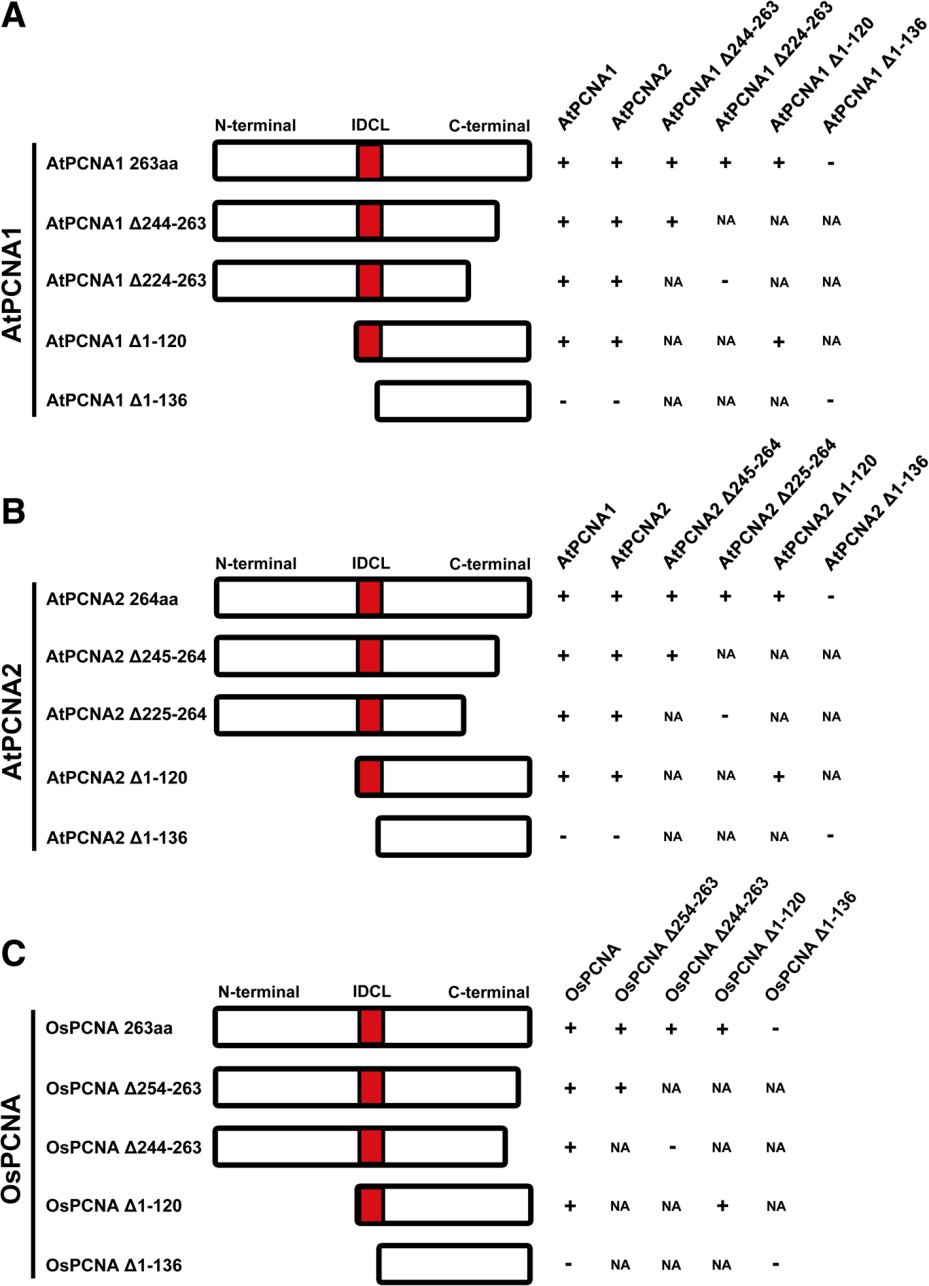
Summary of the regions required for dimerization of OsPCNA and AtPCNA1/2. **a**-**b** Schematic diagram of interaction between PCNA1/2 and its truncated protein in *Arabidopsis*. **c** Schematic diagram of interaction between PCNA and its truncated protein in rice. The symbol “+” means that direct interactions exist between these proteins, while the symbol “-” indicates that no interactions exist between these proteins

In *Arabidopsis*, it has been demonstrated that AtPCNA1 and AtPCNA2 could interact with each other and form four kinds of homotrimer or heterotrimer [31, 32]. Since PCNA is a ring-shaped complex composed of three monomer proteins arranged as a head-to-tail manner, the same truncated PCNA variants were fused to N- or C-terminus of the YFP to identify the regions of PCNA required for the formation of *Arabidopsis* PCNA clamp. When deleting the 20 aa of AtPCNA1 C-terminal, the interactions between it and full-length AtPCNA1 or AtPCNA2 were not affected, so was the interaction with AtPCNA1 Δ244–263 (Fig. 5a; Additional file 5a-c). When deleting the 40 aa of AtPCNA1 C-terminal, it no longer interact with itself, indicating that the region between 225 to 244 aa of AtPCNA1 is responsible for the assemble of PCNA heterodimer or homodimer (Additional file 5d-f). On the other hand, we found that when the 1 to 120 aa of AtPCNA1 N-terminal was truncated, the interactions between AtPCNA1, AtPCNA2, and itself were not affected (Additional file 5 g-i). When deleting the 1 to 136 aa of AtPCNA1 N-terminal, the interactions between AtPCNA1, AtPCNA2, and itself all disappeared (Additional file 5j-l), suggesting that the region between 121 to 136 aa of AtPCNA1 is also required for the formation of PCNA heterodimer or homodimer. Similarly, we found that the regions between 225 to 244 aa and 121 to 136 aa of AtPCNA2 are indispensable for the formation of the PCNA clamp (Additional file 5 m-x).

Afterwards, we investigated the regions required for formation of the OsPCNA homodimer using the truncated variants of OsPCNA. The results showed that obvious YFP signals were accumulated in the cells co-transformed with OsPCNA-YFP^N^ and OsPCNA-YFP^C^, indicating that the OsPCNA monomer could interact with each other (Fig. 5c). Then we found that deleting the 254 to 263 aa of OsPCNA C-terminal did not affect the interactions between PCNA and its monomers. When 20 aa of its C-terminal was truncated, although the interaction between it and the integrated OsPCNA was not affected, the homodimer could not be formed. This indicated that the region between 245 to 254 aa of OsPCNA C-terminal is dispensable for the interactions between PCNA and its monomers. On the other hand, The OsPCNA Δ1–120 and OsPCNA Δ1–136 that lacked 120 aa and 136aa in the N-terminal, could not interact with itself and the full-length OsPCNA, suggesting that the region between 121 to 136 aa of OsPCNA is required for the formation of homodimer (Additional file 6). In summary, the IDCL domain and C-terminal of PCNA protein was required for interactions with its monomers in *Arabidopsis* and rice. Previous study on crystal structure of *Arabidopsis* PCNA indicated that the β-sheets (β8 and β13) from two adjacent PCNA monomers sequenced FESPTQDKIADFEMKL and DIGTANIVLRQNTT interact directly. The two β-sheets are neither at the N-terminal nor C-terminal of PCNA, which is not consistent with our results [31]. However, it has been suggested that the amino acid stretches from N- and C-terminal end of PCNA may be crucial to maintain its native structure [38]. This may explain why the variants no longer interact with each other when the C-terminal region of PCNA was truncated in *Arabidopsis* and rice (Fig. 5). Moreover, we conclude that the IDCL domain of PCNA contribute more to maintain its structure than the N-terminal.

### Essential regions of PCNA which mediate the interactions with RFC subunits in *Arabidopsis* and rice

**Fig. 6:**
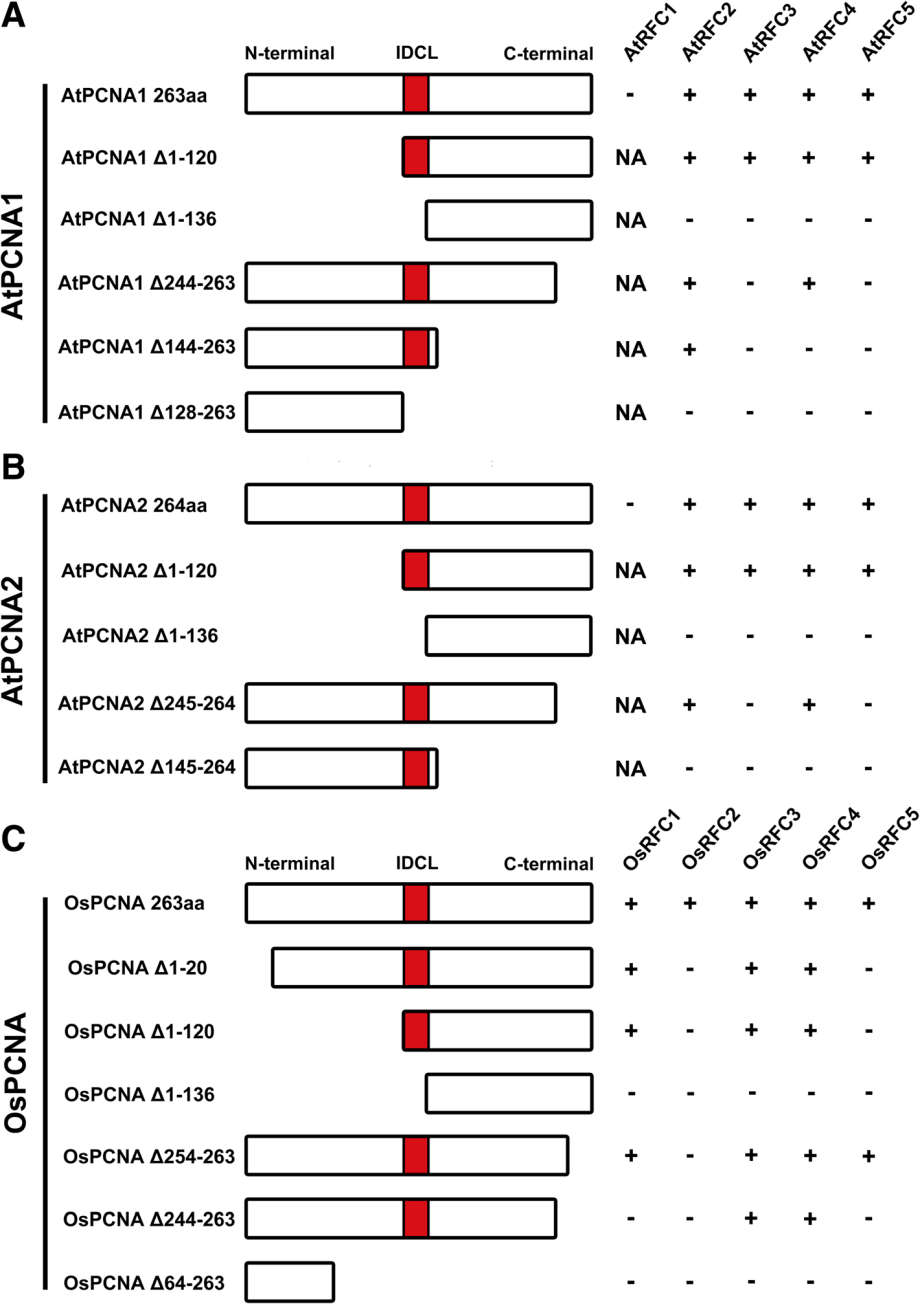
Summary of interactions between the truncated PCNA proteins and RFC subunits in *Arabidopsis* and rice. **a** Schematic diagrams of the regions required for AtPCNA1 to interact with AtRFC1/2/3/4/5. **b** Schematic diagrams of the regions required for AtPCNA2 to interact with AtRFC1/2/3/4/5. **c** Schematic diagrams of the regions required for OsPCNA to interact with OsRFC1/2/3/4/5. The symbol “+” means that direct interactions exist between these proteins, while the symbol “-” indicates that no interactions exist between these proteins

To identify the regions of PCNA responsible for interactions with RFC subunits in *Arabidopsis* and rice, a series of truncated PCNA proteins were fused with N- or C-terminal of the YFP and used in the BiFC assay (Fig. 6). The results showed that AtPCNA1–120 did not affect the interactions with AtRFC2/3/4/5 (Additional file 7a-d). However, AtPCNA1 Δ1–136, with another 16 aa deleted in its N-terminal, could not bind AtRFC2/3/4/5 (Additional file 7e-h), suggesting that the IDCL domain of AtPCNA1 was required for binding RFC complex. On the other hand, we found that a deletion of 20 aa in AtPCNA1 C-terminal did not affect its interactions with AtRFC2/4 (Additional file 7i and k), while the interactions with AtRFC3/5 disappeared (Additional file 7j and l). An additional deletion of 100 aa in its C-terminal did not support the interaction with AtRFC3/4/5 (Additional file 7n-p), while retained the ability of binding AtRFC2 (Additional file 7m). When the 128 to 263 aa of AtPCNA1 including the IDCL domain was deleted, no fluorescent signal could be detected in the cells co-expressing AtPCNA1 Δ128–263-YFP^N^ and AtRFC2/3/4/5-YFP^C^ (Additional file 7q-t). All these results indicated that the region within amino acid 128 to 263 aa of AtPCNA1 mediated its interactions with AtRFC2/3/4/5.

The deletion analysis of AtPCNA2 was also performed (Fig. 6b; Additional file 8). The results showed that the N-terminal deletions of AtPCNA1 did not affect the interactions with AtRFC2/3/4/5 (Additional file 8a-d). But, when the 1 to 136 aa of AtPCNA2 N-terminal including the IDCL domain was deleted, the interactions between AtPCNA2 and AtRFC2/3/4/5 disappeared (Additional file 8e-h), suggesting the IDCL domain is indispensable for binding RFC complex. Meanwhile, we found that a deletion of 20 aa in AtPCNA2 C-terminal retained its interactions with AtRFC2/4 (Additional file 8i and k), but did not support the interactions with AtRFC3/5 (Additional file 8j and l). AtPCNA2 Δ145–264, with an additional deletion of 100 aa in its C-terminal, did not interact with AtRFC2/3/4/5 (Additional file 8m-p). All these results indicated that the region within amino acid 121 to 264 aa of AtPCNA2 mediated its interactions with AtRFC2/3/4/5.

**Fig. 7:**
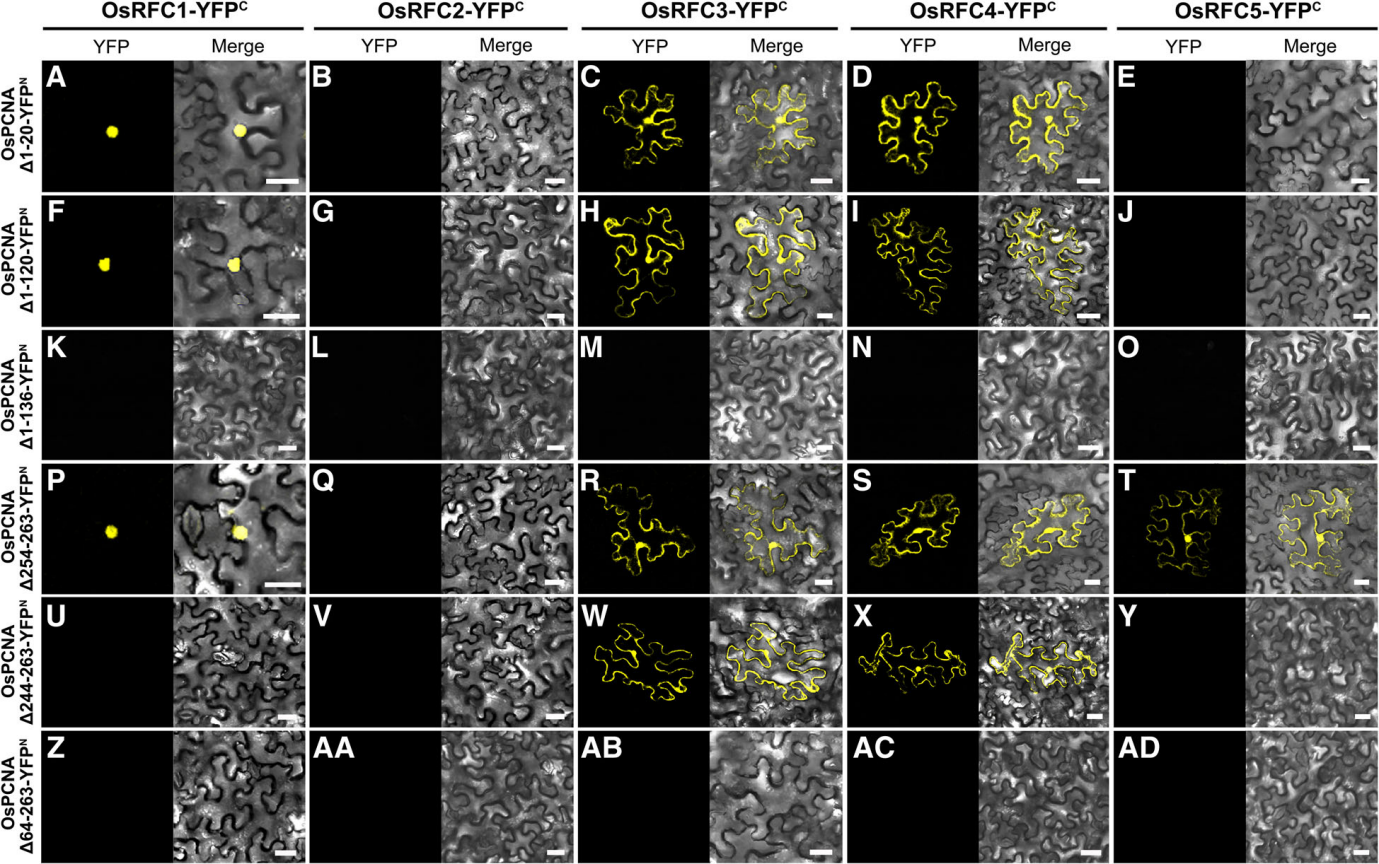
BiFC assays between the truncated OsPCNA and OsRFC1/2/3/4/5 proteins. **a**-**e** Interactions between the truncated OsPCNA Δ1–20 and OsRFC1/2/3/4/5. **f**-**j** Interactions between the truncated OsPCNA Δ1–120 and OsRFC1/2/3/4/5. **k**-**o** Interactions between the truncated OsPCNA Δ1–136 and OsRFC1/2/3/4/5. **p**-**t** Interactions between the truncated OsPCNA Δ254–263 and OsRFC1/2/3/4/5. **u**-**y** Interactions between the truncated OsPCNA Δ244–263 and OsRFC1/2/3/4/5. **z**-ad Interactions between the truncated OsPCNA Δ64–263 and OsRFC1/2/3/4/5. Confocal images of tobacco leaf cells transiently-expressed indicated fusion proteins. YFP^C^, the C-terminal fragment of YFP (156–239 aa); YFP^N^, the N-terminal fragment of YFP (1–155 aa). Bars = 50 μm

Similar experiments were performed on deletion variants of the OsPCNA (Figs. 6c and 7). When OsPCNA had a deletion of 1 to 20 aa in its N-terminal, stable YFP signals were accumulated in tobacco epidermal cells co-transformed OsPCNA Δ1–20-YFP^N^ and OsRFC1/3/4-YFP^C^ (Fig. 7a, c, and d), while no YFP signal was detected in OsPCNA Δ1–20-YFP^N^ and OsRFC2/5-YFP^C^ (Fig. 7b and e). This suggested that 1 to 20 aa of OsPCNA is dispensable for binding OsRFC2 and OsRFC5. OsPCNA Δ1–120 that an additional 100 aa in OsPCNA N-terminal still supported interactions with OsRFC1/3/4 (Fig. 7f, h, and i). When the 1 to 136 aa of OsPCNA was truncated, the variant did not interact with OsRFC1/2/3/4/5 (Fig. 7k-o), suggesting that the IDCL domain of OsPCNA is dispensable for interacting with OsRFC1/3/4. OsPCNA Δ254–263, which had a deletion of 10 aa in OsPCNA C-terminal, retained its interactions with OsRFC1/3/4/5 (Fig. 7p, r-t), but could not support the interaction with OsRFC2 (Fig. 7q). OsPCNA Δ244–263, which possessed an additional deletion of 10 aa in C-terminal, did not interact with OsRFC1/2/5 (Fig. 7u, v, and y), but still could bind OsRFC3/4 (Fig. 7w and x). When the 64 to 263 aa of OsPCNA was deleted, the interactions between OsPCNA and OsRFC1/2/3/4/5 all disappeared (Fig. 7z-ad). These results suggested the IDCL domain, C-terminal, and N-terminal of OsPCNA are all required for binding OsRFC1/2/3/4/5. In summary, the IDCL domain and C-terminal are required for interaction with RFC complex in *Arabidopsis* and rice, whereas the N-terminal of OsPCNA are dispensable for binding OsRFC2 and OsRFC5.

## Discussion

### Differences may exist between the *Arabidopsis* and rice RFC complex in interacting PCNA

Previous study reported that at least three RFC subunits RFC1/3/4 can directly bind the closed PCNA clamp in RFC-PCNA complex [8]. Other researches showed that RFC1/2/4 single subunit could specifically bind the C-terminal of PCNA [36, 37]. In this study, we found that AtPCNA1 and AtPCNA2 could only interact with AtRFC2/3/4/5 subunits (Figs. 1 and 2), which is not consistent with the above results that PCNA could interact with RFC1 [8, 36, 37]. On the other hand, stable interactions were observed between OsPCNA and OsRFC1/2/3/4/5 (Fig. 1b; Fig. 2n-r). These results suggested that there are obvious differences between RFC complex in binding PCNA, and AtRFC1 may not interact directly with PCNA when recognizing and loading it in *Arabidopsis*. There might be three explanations for this result. One possibility is that the interactions between AtRFC1 and AtPCNA1/2 are too weak to be detected by Y2H and BiFC methods. Another explanation is the interactions between AtRFC1 and AtPCNA1/2 require the participation of other one or more RFC subunits. The third possibility is that other proteins might replace AtRFC1 when recognizing and loading PCNA although this is not very likely. Interestingly, stable YFP signals were observed when we tested the combination of OsRFC1-YFP^N^ and AtPCNA1/2- YFP^C^ while no fluorescent signals were accumulated in cells co-expressing AtRFC1-YFP^N^ and OsPCNA-YFP^C^ (Fig. 3a, f, and k). This suggested that *Arabidopsis* PCNA has the ability to bind RFC1 and the lack of interaction between AtRFC1 and AtPCNA1/2 was attributed to the RFC1 partner. Moreover, we found that AtRFC1 interact with AtRFC2/3/4/5 only when all of the five RFC subunits exist at the same time in our previous work [39]. In yeast, four catalytic ATPase sites are located at the RFC5/2, RFC2/3, RFC3/4, and RFC4/1 subunit interfaces [8, 40].

Mutational studies indicated that only three of ATP sites are needed for PCNA clamp loading; the ATP site of RFC1 is not essential for clamp loading [9]. Results from another research also demonstrated that RFC1 is not required for PCNA opening and RFC2/3/4/5 and RFC2/5 subassemblies are capable of opening and unloading PCNA from circular DNA [41]. The results that the conformation of RFC complex and PCNA clamp change greatly in the process of binding sliding clamp and loading it onto the primer-template sites, which also provides the possibility that AtRFC1 may not interact consistently with AtPCNA1/2 [42]. Moreover, we noticed that the C-terminal of *Arabidopsis* RFC2/3/4/5 is essential for binding PCNA, which is not consistent with the situation in yeast, where the N-terminal of RFC subunits attribute to interact with the PCNA ring [8]. However, the regions of rice RFC1/2/3/4/5 required for interacting with PCNA are closer to their N-terminal than C-terminal (Fig. 4), which is different from the results from *Arabidopsis*. The region for rice RFC1 to bind PCNA (641 to 722 aa, Fig. 4) is similar with that of human RFC1 (481 to 728 aa) [31, 43]. Thus, the results of rice seem to be more consistent with the previous conclusions. A general motif governing PCNA-protein interactions is the PIP (PCNA-interacting protein) box that has a conserved sequence Q-x-x-J-x-x-w-w, in which J is a moderately hydrophobic amino acid (L, V, I, or M) and w is an aromatic residue (Y or F) [23, 44]. We failed to identify the putative PIP-BOX in all *Arabidopsis* and rice RFC subunits, nor can we find any similar sequence of APIM (AlkB homolog 2 PCNA-interacting motif), a PCNA-interacting motif widespread among DNA repair proteins and is defined as K/R-F/Y/W-L/I/V/A-L/I/V/A-K/R [23]. It is not clear whether these plant RFC subunits possess other kinds of PIP domains for binding PCNA. Since no PCNA-binding domain of single RFC protein except RFC1 has been identified and confirmed, further research is needed to figure out the exact roles of individual RFC subunit in recognizing and binding PCNA.

### The interactional patterns of RFC-PCNA complex and PCNA clamp are conserved between *Arabidopsis* and rice

Previous studies have shown that RFC complex is able to protect the C-terminal but not the N-terminal region of human PCNA from phosphorylation, suggesting that RFC subunits interact with the C-terminal of PCNA [25, 37]. It has also been proved that the RFC3 subunit in human could interact independently with the C-terminal of PCNA and the RFC1 subunit in *Drosophila melanogaster* interacted similarly with the human PCNA, indicating that the interactions between RFC and PCNA is conserved among eukaryotes [37]. The IDCL domain is a major interaction site for various PCNA-binding proteins involved in DNA replication and repair, including polymerases Polδ, LIG1 (DNA ligase 1), FEN1 (flap endonuclease 1), CDK2 (cyclin dependent kinase 2), cyclin D, and so on [26, 45]. Most of the PCNA-binding proteins contain a PIP motif, indicating that these proteins might bind to the same sites on PCNA ring [43, 46].

To identify which domain is required for the formation of RFC-PCNA complex, a series of truncated PCNA proteins were used in the BiFC assay. We found that IDCL domain and C-terminal regions of *Arabidopsis* PCNA1 and 2 are required for binding RFC subunits (Fig. 6), which is consistent with the previous studies. Moreover, AtPCNA1 and 2 exhibited nearly no differences in interacting RFC proteins except that AtRFC2 bind closer to C-terminal of AtPCNA2 than AtPCNA1 (Figs. 5 and 6; Additional files 5, 7, and 8). On the other hand, the rice PCNA binds OsRFC2 and OsRFC5 through its N-terminal and C-terminal (Fig. 6c), indicating that these two RFC subunits probably be located in the joint between two PCNA monomers. The OsRFC1 binds to the C-terminal of OsPCNA, while OsRFC3 and OsRFC4 are located closer to the N-terminal and IDCL domain of OsPCNA (Fig. 6c). Overall, our results are consistent with previous conclusions that the ring-shaped PCNA complex is arranged as a head-to-tail manner and RFC subunits could be located in different domains of the PCNA monomer [8, 47].

### There are probably two or more PCNA trimers in *Arabidopsis*

Previous studies on protein-protein interactions between *Arabidopsis* PCNA 1 and 2 indicated that they could form four kinds of homo- or hetero-trimeric complexes in vitro [31, 32]. In this study, we found that AtPCNA1 and 2 exhibit only a few differences in binding RFC subunits and interacting with its monomeric protein (Figs. 5 and 6), suggesting their functions probably redundant. So why *Arabidopsis* possesses two highly similar PCNA proteins, while yeast, rice and human only have one? One explanation for this is that the gene dosage of PCNA and its expression level need to match that of other DNA replication proteins. Thus the two PCNA proteins act as backups to each other to prevent the serious consequences of protein dysfunction, which is particularly important for proteins involved in DNA replication or repair. In fact, PCNA is not the only replication factor who has another homologous protein in *Arabidopsis*. If one of the AtCDT1a/b fails to work, the other protein will work to assure the genome stability [48, 49]. Another possible explanation is that AtPCNA1 and AtPCNA2 can form different kinds of PCNA rings for different roles. As it has been reported that AtPCNA2, but not AtPCNA1, could functionally interact with the *Arabidopsis* translesion DNA polymerase η and λ [33, 34]. Taken together, our presented data are one of the milestones before uncovering the functional relevance of identified *Arabidopsis* PCNA complexes, especially in DNA replication and cell cycle control.

## Conclusions

In this study, we investigated the interaction details between PCNA and RFC subunits of *Arabidopsis* and rice via employing Y2H method and BiFC techniques. These results indicated that *Arabidopsis* and rice PCNAs are highly conserved in sequence, structure and pattern of interacting with other PCNA monomer. Nevertheless, there are significant differences between the *Arabidopsis* and rice RFC subunits in binding PCNA. Since AtRFC1 lack the ability to bind AtPCNA1 or AtPCNA2 directly and the PCNA-binding domains of *Arabidopsis* RFC2/3/4/5 subunits located at their C-terminal, whereas these domains are closer to the N-terminal in rice. Moreover, the C-terminal and IDCL domain of *Arabidopsis* and rice PCNAs contribute to the interactions with RFC subunits although the motif of OsPCNA for binding OsRFC3 and OsRFC4 located at its N-terminal and independently from the IDCL domains. Our data strengthened the knowledge to understand the interaction relationship between the RFC and PCNA complex and provided details for further revealing the biological functions of PCNA clamp in higher plants.

## Methods

### Plant materials and growth conditions

*Nicotiana benthamiana* seeds were provided by College of Life Sciences, Wuhan University, China. The *Nicotiana benthamiana* used in this study were grown in the greenhouse under artificial light to maintain a 16 h light and 8 h darkness photoperiod at 22 ± 2 °C. For the BiFC experiments, the leaves of 5-week-old plants were used.

### Phylogenetic analysis

The protein sequences of PCNA1/2 in *Arabidopsis* and PCNA in rice were identified through using the *Arabidopsis* Information Resource (TAIR) database (https://www.arabidopsis.org/) and the National Center for Biotechnology Information (NCBI) database (https://www.ncbi.nlm.nih.gov/), respectively. The sequences of AtPCNA1/2 and OsPCNA were used to search for PCNA homologs in other species. Multiple sequence alignment was performed using the DNAMAN software. A neighbor-joining tree was constructed using the MEGA4 software.

### Quantitative real-time PCR

Total RNA from various tissues was extracted by RNAiso Plus (TaKaRa, Japan).

Quantitative Real-Time PCR (qRT-PCR) was carried out using TransStart Eco qPCR SuperMix (TransGen, China) in a BIO-RAD CFX Connect machine (BIO-RAD, USA). At least three biological replicates were performed for each gene, and at least three technical replicates were performed for each biological replicate. The method for analyzing the relative expression levels is the △△Ct method [50], and the *GAPDH* and *Actin* were applied as reference genes for *Arabidopsis* and rice PCNA genes in qRT-PCR analysis, respectively.

### Construction of vectors for yeast-two-hybrid and BiFC analysis

To construct the vectors for Y2H analysis, the full-length open reading frames (ORFs) of *AtRFC1/2/3/4/5*, *OsRFC1/2/3/4/5, AtPCNA1/2* and *OsPCNA* with stop codon were amplified with the help of KOD-Plus-Neo polymerase (TOYOBO, http://www.toyobo-global.com) using specific primers (Additional file 9). Then, the PCR products were purified using an AxyPrep™ PCR Cleanup Kit (Axygen, http://www.axygen.com.cn) and cloned into the *pGADT7* and *pGBKT7* vectors, respectively. Similarly, the full-length open reading frames (ORFs) of *AtRFC1/2/3/4/5*, *OsRFC1/2/3/4/5, AtPCNA1/2* and *OsPCNA* were amplified and cloned into the *pCAMBIA-SPYNE* and *pCAMBIA-SPYCE* vectors for BiFC assay.

### Yeast-two-hybrid analysis

A yeast-two-hybrid system (Clontech, www.takarabio.com) was used to test interactions between AtPCNA1/2 and AtRFC1/2/3/4/5, OsPCNA and OsRFC1/2/3/4/5 proteins. The AH109 yeast strain was transformed with appropriate combinations of bait and prey plasmids along with negative control vectors. After transformation, the yeast cells were transferred onto SD-Leu-Trp selection plates followed by a 3-day incubation at 28 °C. The transformed cells were plated on an SD-Leu-Trp-His-Ade solid medium, and incubated for 7 days at 28 °C before analysis.

### BiFC assay

The BiFC analysis was performed as described previously [51]. Fluorescent signals of YFP were observed under an Olympus FluoView FV1000 confocal microscope to determine whether the two designate proteins could interact with each other. Under the confocal microscope (OLYMPUS Fluoview 1000), YFP signal was excited with an argon laser at a wavelength of 515 nm and emissed at wavelength of between 505 nm and 530 nm.

### Accession numbers

The accession numbers of genes used in this study are: AtRFC1 (At5g22010), AtRFC2 (At1g63160), AtRFC3 (At1g77470), AtRFC4 (At1g21690), AtRFC5 (At5g27740), AtPCNA1 (At1g07370), AtPCNA2 (At2g29570), OsRFC1 (Os11g0572100), OsRFC2 (Os12g0176500), OsRFC3 (Os02g0775200), OsRFC4 (Os04g0569000), OsRFC5 (Os03g0792600), OsPCNA (Os02g0805200). The accession numbers of proteins used in this study are: AtPCNA1 (NP_172217.1), AtPCNA2 (NP_180517.1), OsPCNA (XP_015627245.1), HsPCNA (CAG38740.1), ScPCNA (NP_009645.1), ZmPCNA (NP_001105461.1), BnPCNA (NP_001303041.1), CePCNA (NP_500466.3), DmPCNA (XP_002091715.2), DrPCNA (NP_571479.2), GhPCNA (XP_016740519.1), GmPCNA (NP_001241553.1), MmPCNA (NP_035175.1), NbPCNA (CAA10108.1), and PtPCNA (XP_002298328.1).

## Additional files

Additional file 1: Full-length amino acid sequences alignment and phylogenetic analysis of PCNA homologues in eukaryotes. At, *Arabidopsis thaliana*; Hs, *Homo sapiens*; Mu, *Mus musculus*; Os, *Oryza sativa*; Ce, *Caenorhabditis elegans*; Sc, *Saccharomyces cerevisiae*; Pt, *Populus trichocarpa*; Gm, *Glycine max*; Zm, *Zea mays*; Gh, *Gossypium hirsutum*; Dm, *Drosophila melanogaster*; Dr., *Danio rerio*; Bn, *Brassica napus*; Nb, *Nicotiana tabacum*. The AtPCNA1/2 and OsPCNA are highlighted by box and circle. (JPG 9218 kb)

Additional file 2: Temporal and spatial expression of *AtPCNA1/2* and *OsPCNA* genes. (a-b) Expression levels of the AtPCNA1/2 genes in various organs by qPCR assay. (c) Expression levels of the OsPCNA gene in various organs by qPCR assay. Abbreviations: Sd, seedling; R, root; S, stem; L, leaf; In, inflorescence; 1DSi, 1 DAP silique; 2DSi, 2 DAP silique; 3DSi, 3 DAP silique; P1, panicles at 0-3 cm. (JPG 2707 kb)

Additional file 3: BiFC assays between AtPCNA1/2 and the truncated AtRFC2/3/4/5 proteins. (a-f) Interactions between the truncated AtRFC2 and AtPCNA1/2. (g-j) Interactions between the truncated AtRFC3 and AtPCNA1/2. (k-p) Interactions between the truncated AtRFC4 and AtPCNA1/2. (q-t) Interactions between the truncated AtRFC5 and AtPCNA1/2. Confocal images of tobacco leaf cells transiently-expressed indicated fusion proteins. YFP^C^, the C-terminal fragment of YFP (156–239 aa); YFP^N^, the N-terminal fragment of YFP (1–155 aa). Bars = 50 μm. (JPG 8427 kb)

Additional file 4: BiFC assays between OsPCNA and the truncated OsRFC1/2/3/4/5 proteins. (a-c) Interactions between the truncated OsRFC1 and OsPCNA. (d-f) Interactions between the truncated OsRFC2 and OsPCNA. (g-j) Interactions between the truncated OsRFC3 and OsPCNA. (k-m) Interactions between the truncated OsRFC4 and OsPCNA. (n-p) Interactions between the truncated OsRFC5 and OsPCNA. Confocal images of tobacco leaf cells transiently-expressed indicated fusion proteins. YFP^C^, the C-terminal fragment of YFP (156–239 aa); YFP^N^, the N-terminal fragment of YFP (1–155 aa). Bars = 50 μm. (JPG 2448 kb)

Additional file 5: Regions required for dimerization of AtPCNA1 and AtPCNA2. (a-c) Interactions between the truncated AtPCNA1 Δ244–263 proteins and AtPCNA1/2. (d-f) Interactions between the truncated AtPCNA1 Δ224–263 proteins and AtPCNA1/2. (g-i) Interactions between the truncated AtPCNA1 Δ1–120 proteins and AtPCNA1/2. (j-l) Interactions between the truncated AtPCNA1 Δ1–136 proteins and AtPCNA1/2. (m-o) Interactions between the truncated AtPCNA2 Δ245–264 proteins and AtPCNA1/2. (p-r) Interactions between the truncated AtPCNA2 Δ225–264 proteins and AtPCNA1/2. (s-u) Interactions between the truncated AtPCNA2 Δ1–120 proteins and AtPCNA1/2. (v-x) Interactions between the truncated AtPCNA2 Δ1–136 proteins and AtPCNA1/2. Confocal images of tobacco leaf cells transiently-expressed indicated fusion proteins. YFP^C^, the C-terminal fragment of YFP (aa 156–239); YFP^N^, the N-terminal fragment of YFP (aa 1–155). Bars = 50 μm. (JPG 10161 kb)

Additional file 6: Regions required for dimerization of OsPCNA. (a-b) OsPCNA can form homodimer. (c-d) Interactions between the truncated OsPCNA Δ254–263 proteins and OsPCNA. (e-f) Interactions between the truncated OsPCNA Δ244–263 proteins and OsPCNA. (g-h) Interactions between the truncated OsPCNA Δ1–120 proteins and OsPCNA. (i-j) Interactions between the truncated OsPCNA Δ1–136 proteins and OsPCNA. Confocal images of tobacco leaf cells transiently-expressed indicated fusion proteins. YFP^C^, the C-terminal fragment of YFP (aa 156–239); YFP^N^, the N-terminal fragment of YFP (aa 1–155). Bars = 50 μm. (JPG 3446 kb)

Additional file 7: BiFC assays between the truncated AtPCNA1 and AtRFC2/3/4/5 proteins. (a-d) Interactions between the truncated AtPCNA1 Δ1–120 and AtRFC2/3/4/5. (e-h) Interactions between the truncated AtPCNA1 Δ1–136 and AtRFC2/3/4/5. (i-l) Interactions between the truncated AtPCNA1 Δ244–263 and AtRFC2/3/4/5. (m-p) Interactions between the truncated AtPCNA1 Δ144–263 and AtRFC2/3/4/5. (q-t) Interactions between the truncated AtPCNA1 Δ128–263 and AtRFC2/3/4/5. Confocal images of tobacco leaf cells transiently-expressed indicated fusion proteins. YFP^C^, the C-terminal fragment of YFP (156–239 aa); YFP^N^, the N-terminal fragment of YFP (1–155 aa). Bars = 50 μm. (JPG 4280 kb)

Additional file 8: BiFC assays between the truncated AtPCNA2 and AtRFC2/3/4/5 proteins. (a-d) Interactions between the truncated AtPCNA2 Δ1–120 and AtRFC2/3/4/5. (e-h) Interactions between the truncated AtPCNA2 Δ1–136 and AtRFC2/3/4/5. (i-l) Interactions between the truncated AtPCNA2 Δ245–264 and AtRFC2/3/4/5. (m-p) Interactions between the truncated AtPCNA2 Δ145–264 and AtRFC2/3/4/5. Confocal images of tobacco leaf cells transiently-expressed indicated fusion proteins. YFP^C^, the C-terminal fragment of YFP (156–239 aa); YFP^N^, the N-terminal fragment of YFP (1–155 aa). Bars = 50 μm. (JPG 3994 kb)

Additional file 9: Primers (5′ to 3′) used in this study. (DOC 101 kb)

## Data Availability

All data can be found within the manuscript and additional files. The datasets used and/or analyzed during the current study are available from the corresponding author on reasonable request.

## Abbreviations

APIM: AlkB homolog 2 PCNA-interacting motif
BiFC: Bimolecular fluorescence complementation
CDC6: Ccell division control protein 6
CDT1: Chromatin licensing and DNA replication factor 1
IDCL: Interdomain connecting loop
NCBI: National center for biotechnology information
ORF: Open reading frames
PCNA: Proliferating cell nuclear antigen
PIP: PCNA-Interacting protein
RFC: Replication factor C
TAIR: The *Arabidopsis* information resource database
Y2H: Yeast-two-hybrid system
