# Supplementary figures and images for: Interactional similarities and differences in the protein complex of PCNA and DNA replication factor C between rice and Arabidopsis
Source: BMC Plant Biol. 2019 Jun 14;19:257. doi: 10.1186/s12870-019-1874-z (PMC6570896; doi:10.1186/s12870-019-1874-z)

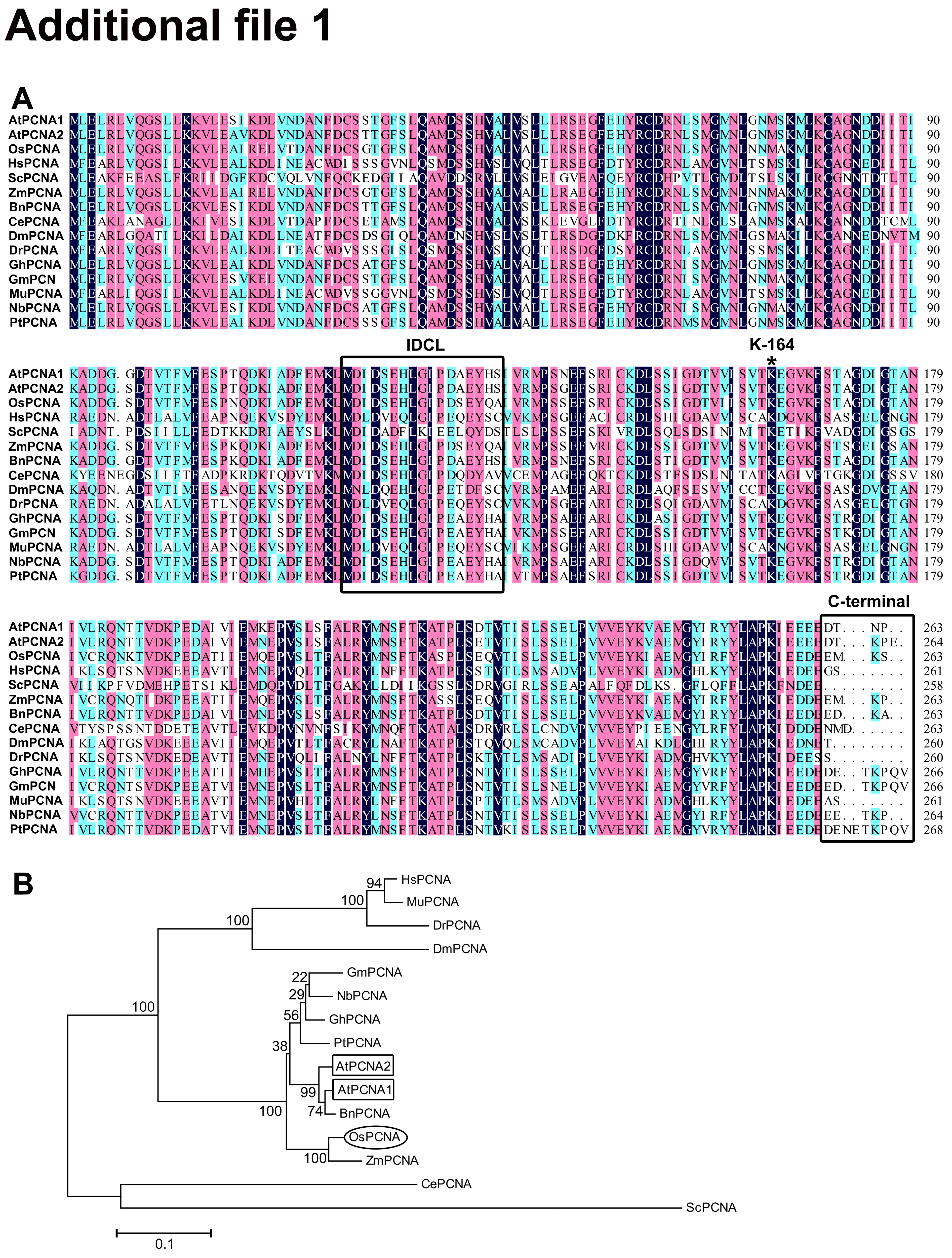

Supplement: Supplementary file 1 — Full-length amino acid sequences alignment and phylogenetic analysis of PCNA homologues in eukaryotes. At, Arabidopsis thaliana; Hs, Homo sapiens; Mu, Mus musculus; Os, Oryza sativa; Ce, Caenorhabditis elegans; Sc, Saccharomyces cerevisiae; Pt, Populus trichocarpa; Gm, Glycine max; Zm, Zea mays; Gh, Gossypium hirsutum; Dm, Drosophila melanogaster; Dr., Danio rerio; Bn, Brassica napus; Nb, Nicotiana tabacum. The AtPCNA1/2 and OsPCNA are highlighted by box and circle. (JPG 9218 kb) [file 12870_2019_1874_MOESM1_ESM.jpg]

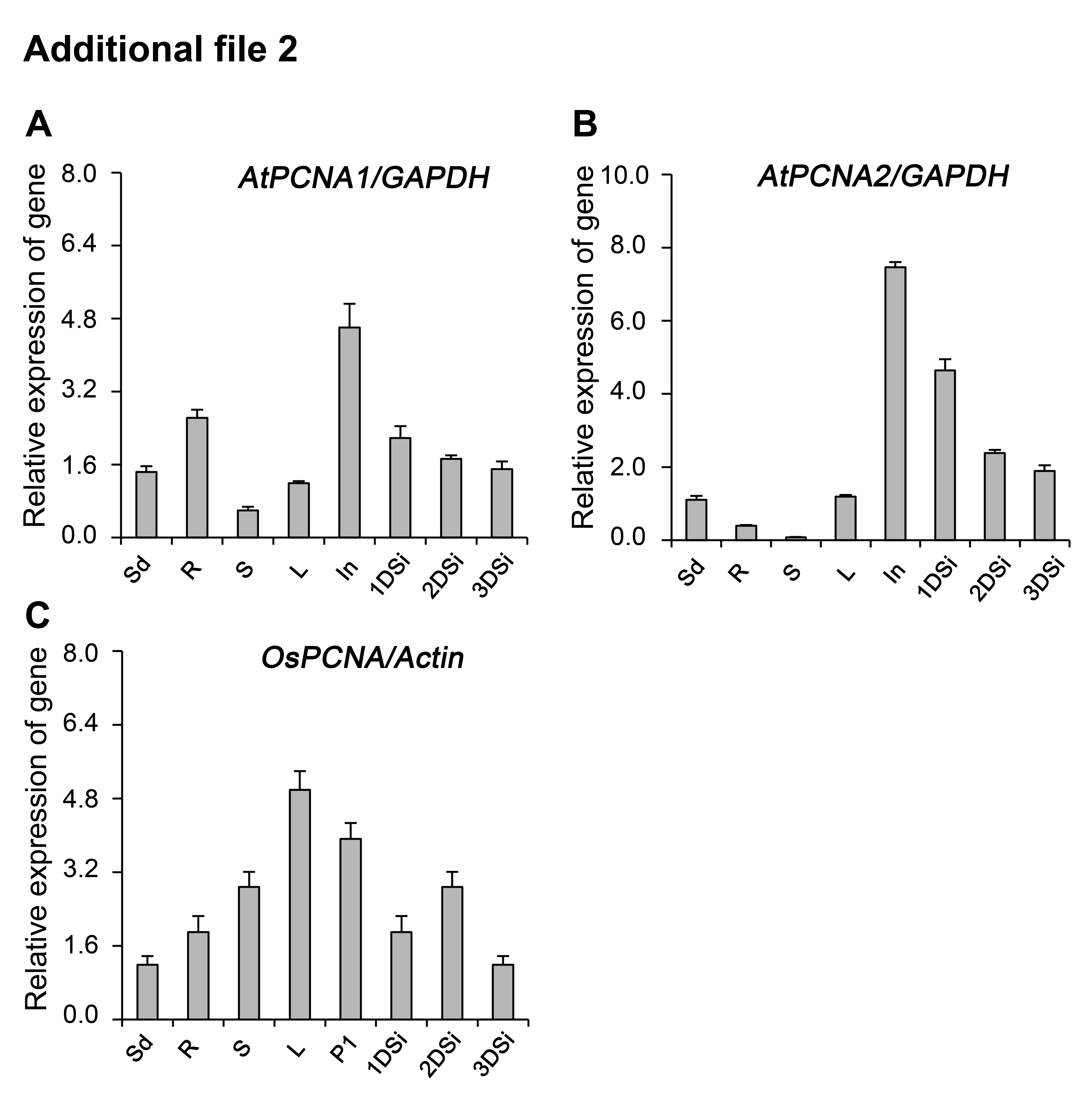

Supplement: Supplementary file 2 — Temporal and spatial expression of AtPCNA1/2 and OsPCNA genes. (a-b) Expression levels of the AtPCNA1/2 genes in various organs by qPCR assay. (c) Expression levels of the OsPCNA gene in various organs by qPCR assay. Abbreviations: Sd, seedling; R, root; S, stem; L, leaf; In, inflorescence; 1DSi, 1 DAP silique; 2DSi, 2 DAP silique; 3DSi, 3 DAP silique; P1, panicles at 0-3 cm. (JPG 2707 kb) [file 12870_2019_1874_MOESM2_ESM.jpg]

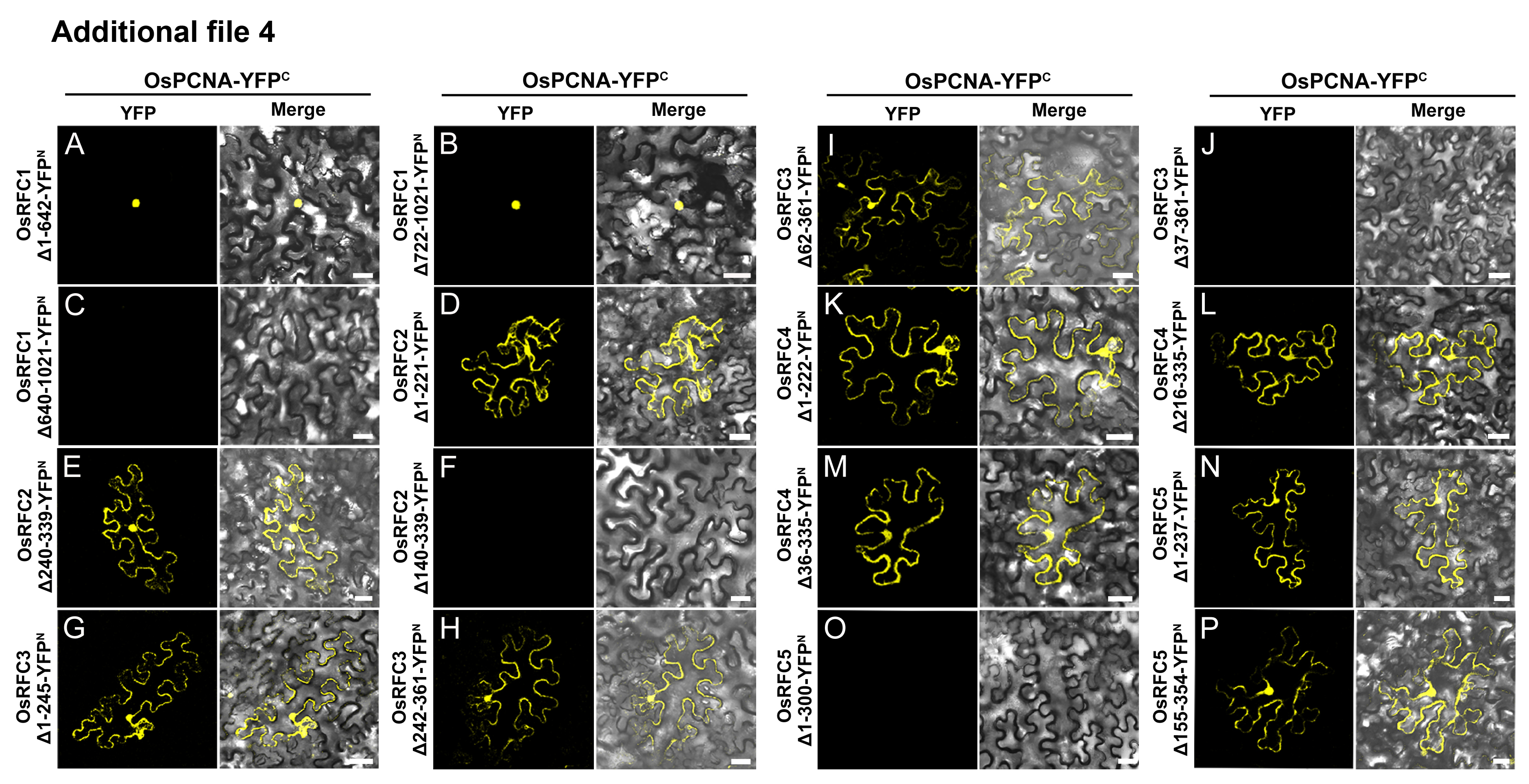

Supplement: Supplementary file 4 — BiFC assays between OsPCNA and the truncated OsRFC1/2/3/4/5 proteins. (a-c) Interactions between the truncated OsRFC1 and OsPCNA. (d-f) Interactions between the truncated OsRFC2 and OsPCNA. (g-j) Interactions between the truncated OsRFC3 and OsPCNA. (k-m) Interactions between the truncated OsRFC4 and OsPCNA. (n-p) Interactions between the truncated OsRFC5 and OsPCNA. Confocal images of tobacco leaf cells transiently-expressed indicated fusion proteins. YFPC, the C-terminal fragment of YFP (156–239 aa); YFPN, the N-terminal fragment of YFP (1–155 aa). Bars = 50 μm. (JPG 2448 kb) [file 12870_2019_1874_MOESM4_ESM.jpg]

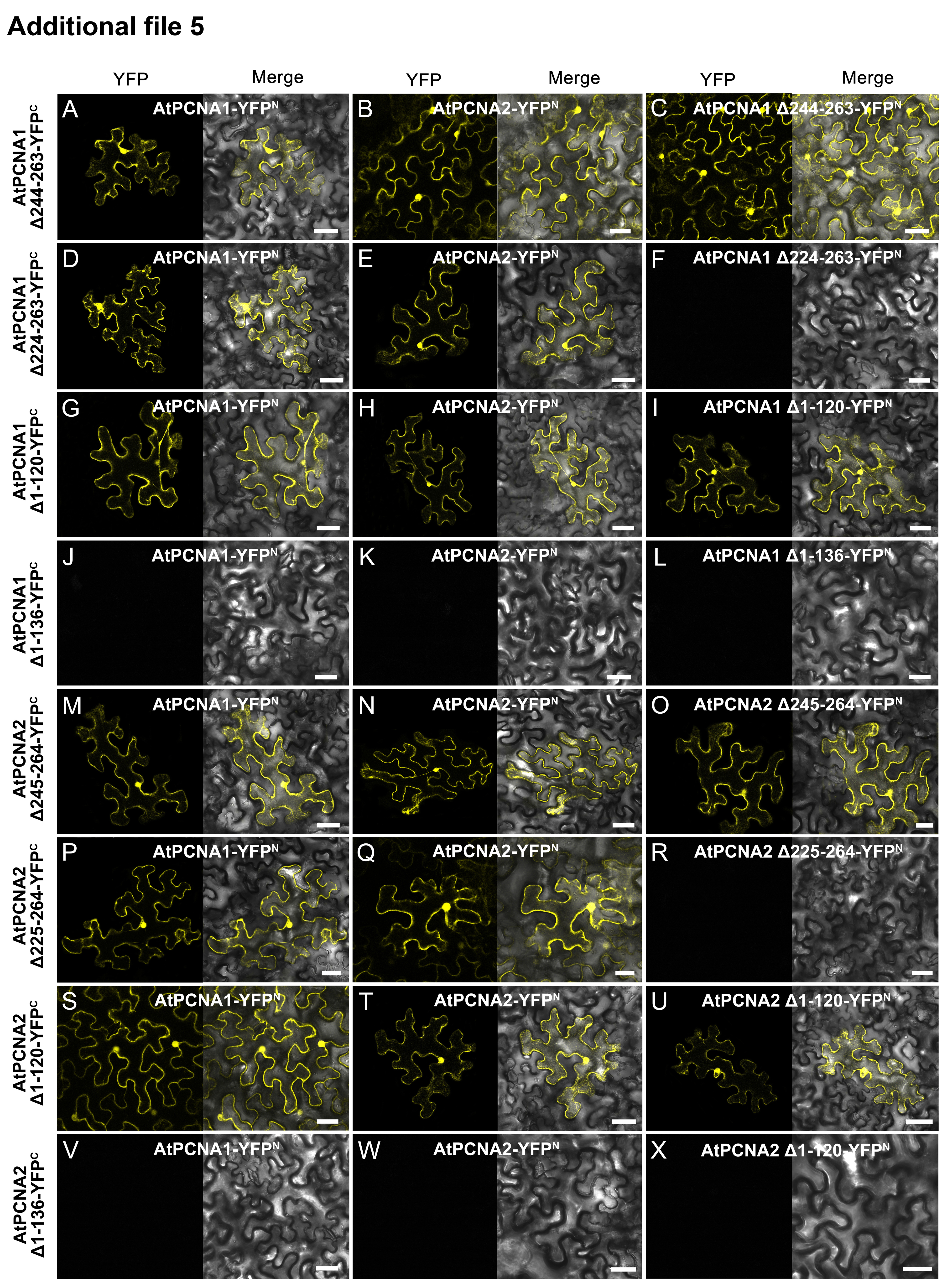

Supplement: Supplementary file 5 — Regions required for dimerization of AtPCNA1 and AtPCNA2. (a-c) Interactions between the truncated AtPCNA1 Δ244–263 proteins and AtPCNA1/2. (d-f) Interactions between the truncated AtPCNA1 Δ224–263 proteins and AtPCNA1/2. (g-i) Interactions between the truncated AtPCNA1 Δ1–120 proteins and AtPCNA1/2. (j-l) Interactions between the truncated AtPCNA1 Δ1–136 proteins and AtPCNA1/2. (m-o) Interactions between the truncated AtPCNA2 Δ245–264 proteins and AtPCNA1/2. (p-r) Interactions between the truncated AtPCNA2 Δ225–264 proteins and AtPCNA1/2. (s-u) Interactions between the truncated AtPCNA2 Δ1–120 proteins and AtPCNA1/2. (v-x) Interactions between the truncated AtPCNA2 Δ1–136 proteins and AtPCNA1/2. Confocal images of tobacco leaf cells transiently-expressed indicated fusion proteins. YFPC, the C-terminal fragment of YFP (aa 156–239); YFPN, the N-terminal fragment of YFP (aa 1–155). Bars = 50 μm. (JPG 10161 kb) [file 12870_2019_1874_MOESM5_ESM.jpg]

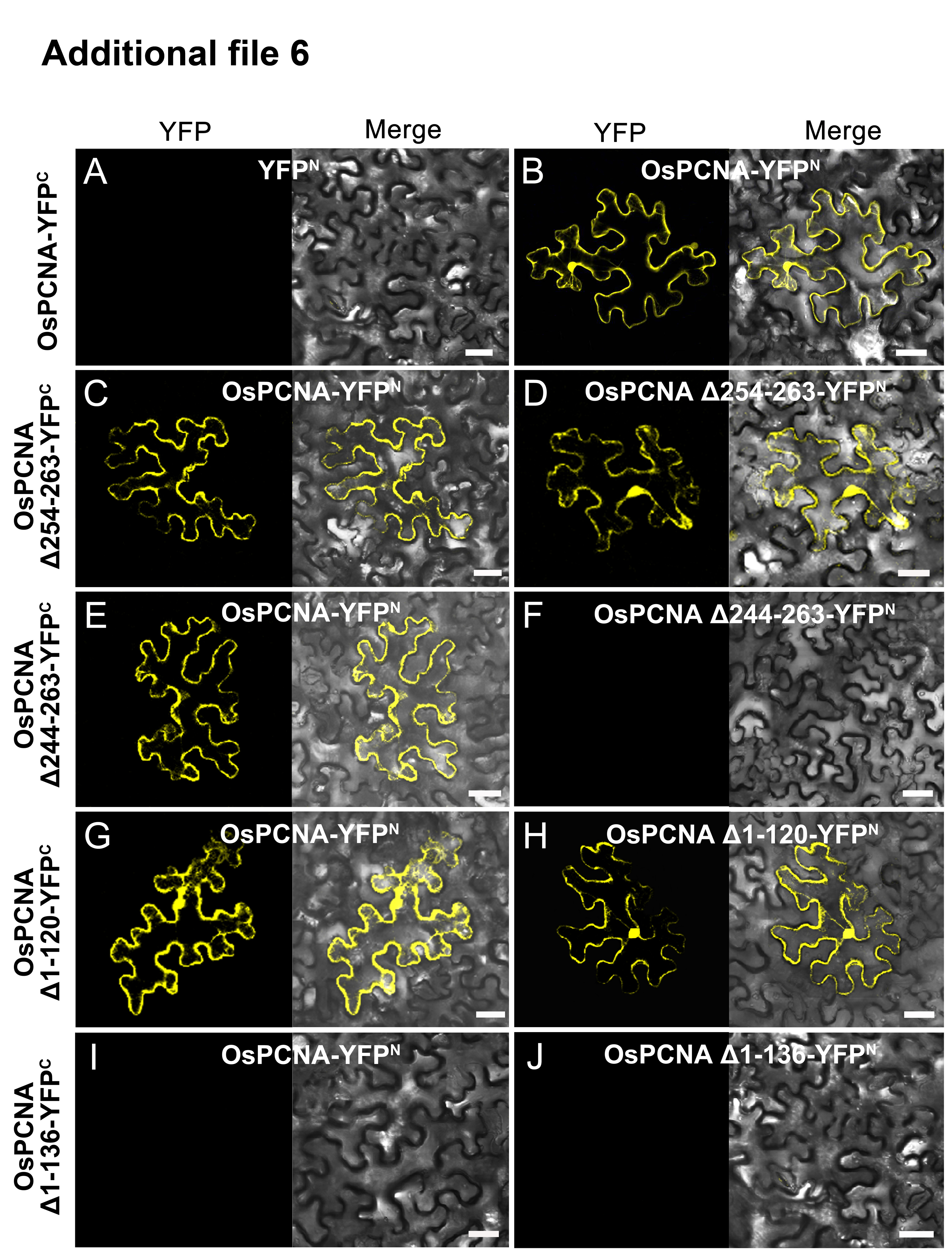

Supplement: Supplementary file 6 — Regions required for dimerization of OsPCNA. (a-b) OsPCNA can form homodimer. (c-d) Interactions between the truncated OsPCNA Δ254–263 proteins and OsPCNA. (e-f) Interactions between the truncated OsPCNA Δ244–263 proteins and OsPCNA. (g-h) Interactions between the truncated OsPCNA Δ1–120 proteins and OsPCNA. (i-j) Interactions between the truncated OsPCNA Δ1–136 proteins and OsPCNA. Confocal images of tobacco leaf cells transiently-expressed indicated fusion proteins. YFPC, the C-terminal fragment of YFP (aa 156–239); YFPN, the N-terminal fragment of YFP (aa 1–155). Bars = 50 μm. (JPG 3446 kb) [file 12870_2019_1874_MOESM6_ESM.jpg]

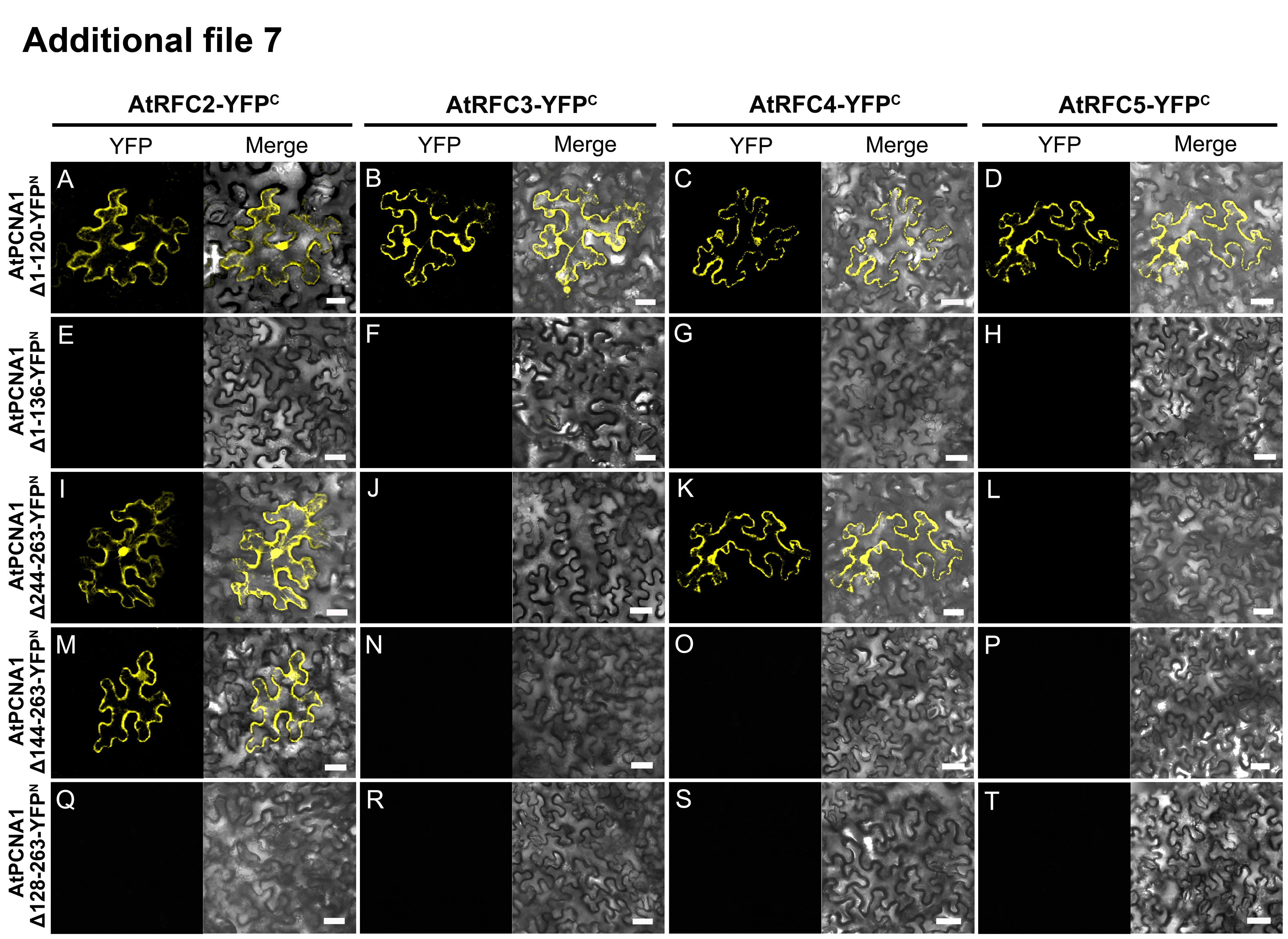

Supplement: Supplementary file 7 — BiFC assays between the truncated AtPCNA1 and AtRFC2/3/4/5 proteins. (a-d) Interactions between the truncated AtPCNA1 Δ1–120 and AtRFC2/3/4/5. (e-h) Interactions between the truncated AtPCNA1 Δ1–136 and AtRFC2/3/4/5. (i-l) Interactions between the truncated AtPCNA1 Δ244–263 and AtRFC2/3/4/5. (m-p) Interactions between the truncated AtPCNA1 Δ144–263 and AtRFC2/3/4/5. (q-t) Interactions between the truncated AtPCNA1 Δ128–263 and AtRFC2/3/4/5. Confocal images of tobacco leaf cells transiently-expressed indicated fusion proteins. YFPC, the C-terminal fragment of YFP (156–239 aa); YFPN, the N-terminal fragment of YFP (1–155 aa). Bars = 50 μm. (JPG 4280 kb) [file 12870_2019_1874_MOESM7_ESM.jpg]

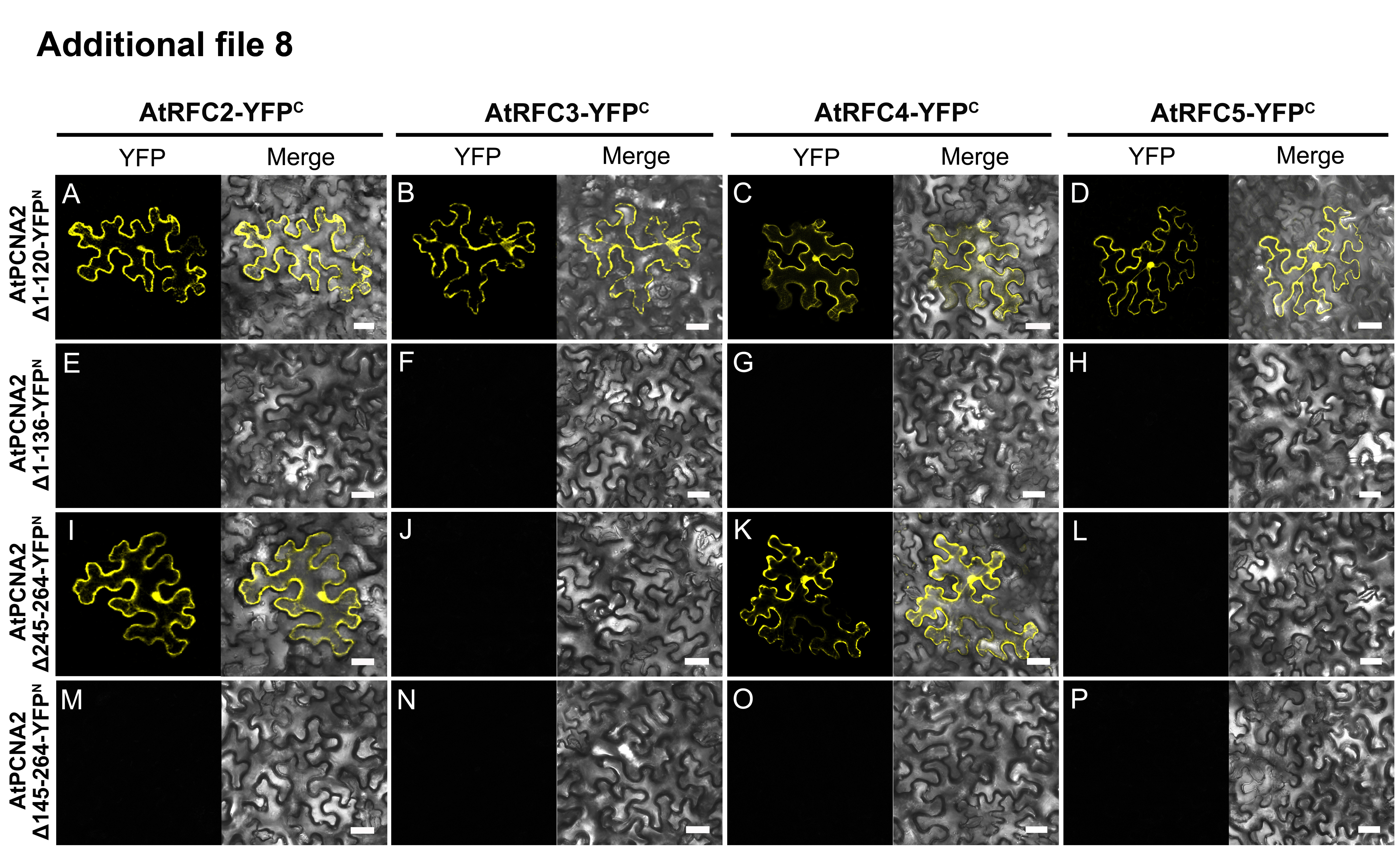

Supplement: Supplementary file 8 — BiFC assays between the truncated AtPCNA2 and AtRFC2/3/4/5 proteins. (a-d) Interactions between the truncated AtPCNA2 Δ1–120 and AtRFC2/3/4/5. (e-h) Interactions between the truncated AtPCNA2 Δ1–136 and AtRFC2/3/4/5. (i-l) Interactions between the truncated AtPCNA2 Δ245–264 and AtRFC2/3/4/5. (m-p) Interactions between the truncated AtPCNA2 Δ145–264 and AtRFC2/3/4/5. Confocal images of tobacco leaf cells transiently-expressed indicated fusion proteins. YFPC, the C-terminal fragment of YFP (156–239 aa); YFPN, the N-terminal fragment of YFP (1–155 aa). Bars = 50 μm. (JPG 3994 kb) [file 12870_2019_1874_MOESM8_ESM.jpg]
