# Supplementary material for: Interactional similarities and differences in the protein complex of PCNA and DNA replication factor C between rice and Arabidopsis
Source: BMC Plant Biol. 2019 Jun 14;19:257. doi: 10.1186/s12870-019-1874-z (PMC6570896; doi:10.1186/s12870-019-1874-z)
Supplement: Supplementary file 9 — Primers (5′ to 3′) used in this study. (DOC 101 kb) [file 12870_2019_1874_MOESM9_ESM.doc]

| Table S1. Primers (5' to 3') used in this study | | | | |
| --- | --- | --- | --- | --- |
| **1.1 Primers for yeast two-hybrid and BiFC** | | | | |
|  | FP | | RP | |
| OsRFC1-AD | cgGAATTCatgtcttcggacatcaggaaat | | cGAGCTCGcctcttcctcttcccacctg | |
| OsRFC1-BK | cgGAATTCatgtcttcggacatcaggaaat | | gcGTCGACGcctcttcctcttcccacctg | |
| OsRFC2-AD/BK | cgGAATTCatggcgccgctcgtgccg | | cgGGATCCcgaagaccagcgtttgtggcatgtc | |
| OsRFC3-AD/BK | cgGAATTCatggcgggagccaccgcc | | cgGGATCCcgtgggcagcagcaaccatagcc | |
| OsRFC4-AD/BK | cgGAATTCatggacgcctccagctcct | | cgGGATCCctgatgcttttgctgtttctctca | |
| OsRFC5-AD/BK | cgGAATTCatgctgtgggtggacaagta | | cgGGATCCcgccaaatgtagagacaagaaatt | |
| OsPCNA-AD/BK | cgGAATTCttggagctgaggcttgtgca | | cgGGATCCccgacttcatttcctcatct | |
| OsRFC1-YC/YN | gcTCTAGAatgtcttcggacatcaggaaat | | gcGTCGACcctcttcctcttcccacctg | |
| OsRFC2-YC/YN | cgGGATCCatggcgccgctcgtgccg | | ggGGTACCgaagaccagcgtttgtggcatgtc | |
| OsRFC3-YC/YN | gcTCTAGAatggcgggagccaccgcc | | cgGGATCCgtgggcagcagcaaccatagcc | |
| OsRFC4-YC/YN | gcTCTAGAatggacgcctccagctcct | | cgGGATCCtgatgcttttgctgtttctctca | |
| OsRFC5-YC/YN | gcTCTAGAatgctgtgggtggacaagta | | cgGGATCCgccaaatgtagagacaagaaatt | |
| OsPCNA-YC/YN | gcGTCGACatgttggagctgaggcttgt | | cggGGTACCcgacttcatttcctcatctt | |
| AtRFC2-AD/BK | cgcGAATTCatggcgtcttcttcatcaac | | agaGGATCCcaggtgctttcgctgtttca | |
| AtRFC3-AD/BK | cgcGAATTCatgttgtgggtcgacaagta | | agaGGATCCccccaaacgttgaaatgagg | |
| AtRFC4-AD/BK | cgcGAATTCatggcgccagttcttcagag | | agaGGATCCcgaagtcttgagccatttctg | |
| AtRFC5-AD/BK | cgcGAATTCatgactgagctaacgtcggc | | agaGGATCCcctttgctgcaccaacaatg | |
| AtPCNA1-AD/BK | agaGGATCCgtatgttggagctacgtcttgt | | cgcGGATCCcgggattagtgtcttcttctt | |
| AtPCNA2-AD/BK | agaGGATCCgtatgttggagcttcgtttagt | | cgcGGATCCcttctggtttggtgtcttctt | |
| AtRFC2-YC/YN | agaGTCGACatggcgtcttcttcatcaac | | cgcGGTACCaggtgctttcgctgtttcac | |
| AtRFC3-YC/YN | agaGTCGACatgactgagctaacgtcggc | | cgcGGTACCctttgctgcaccaacaatga | |
| AtRFC4-YC/YN | agaGTCGACatggcgccagttcttcagag | | cgcGGTACCcactatgatagtctcataag | |
| AtRFC5-YC/YN | cgcTCTAGAatgttgtgggtcgacaagta | | cgcGGATCCcccaaacgttgaaatgagga | |
| AtPCNA1-YC/YN | agaGTCGACatgttggagctacgtcttgt | | cgcGGTACCgggattagtgtcttcttctt | |
| AtPCNA2-YC/YN | agaGGATCCatgttggagcttcgtttagt | | cgcGGTACCttctggtttggtgtcttctt | |
| **1.2 Primers for deletion analysis of RFC and PCNA** | | | | |
|  | | FP | | RP |
| OsRFC1 Δ1-642 | | gcTCTAGAatgctctcccagtccgtggtca | | gcGTCGACcctcttcctcttcccacctg |
| OsRFC1 Δ1-721 | | gcTCTAGAatggttaaaagaatgaattttct | | gcGTCGACcctcttcctcttcccacctg |
| OsRFC1 Δ722-1021 | | gcTCTAGAatgtcttcggacatcaggaaat | | gcGTCGACtccactgtcatccttcccaa |
| OsRFC1 Δ640-1021 | | gcTCTAGAatgtcttcggacatcaggaaat | | gcGTCGACgactgggagaggctcataa |
| OsRFC2 Δ1-221 | | cgGGATCCatgttatttggatcttctattt | | ggGGTACCgaagaccagcgtttgtggcatgtc |
| OsRFC2 Δ320-339 | | cgGGATCCatggcgccgctcgtgccg | | ggGGTACCaagctgtaaatactcatccg |
| OsRFC2 Δ300-339 | | cgGGATCCatggcgccgctcgtgccg | | ggGGTACCacatattcttgccttctgct |
| OsRFC2Δ240-339 | | cgGGATCCatggcgccgctcgtgccg | | ggGGTACCgatagcccctgaaacacta |
| OsRFC2 Δ140-339 | | cgGGATCCatggcgccgctcgtgccg | | ggGGTACCctccatagtacgccttaaagca |
| OsRFC3 Δ1-245 | | gcTCTAGAatggcatctaagcaaataacag | | cgGGATCCgtgggcagcagcaaccatagc |
| OsRFC3 Δ342-361 | | gcTCTAGAatggcgggagccaccgcc | | cgGGATCCctgtaacttgtcattgcaag |
| OsRFC3 Δ322-361 | | gcTCTAGAatggcgggagccaccgcc | | cgGGATCCcagctttatgcgtacatcag |
| OsRFC3 Δ302-361 | | gcTCTAGAatggcgggagccaccgcc | | cgGGATCCtatgatatcaaccaaggcca |
| OsRFC3 Δ62-361 | | gcTCTAGAatggcgggagccaccgcc | | cgGGATCCggtgtcgacgatgtcgcggtg |
| OsRFC4 Δ1-222 | | gcTCTAGAatggggttccgttttgttaatc | | cgGGATCCtgatgcttttgctgtttctctca |
| OsRFC4 Δ216-335 | | gcTCTAGAatggacgcctccagctcct | | cgGGATCCgttcaaagcttgcctcatgt |
| OsRFC4 Δ136-335 | | gcTCTAGAatggacgcctccagctcct | | cgGGATCCcagtgcttgctgtgctcccgat |
| OsRFC4 Δ36-335 | | gcTCTAGAatggacgcctccagctcct | | cgGGATCCggagttgccgccgacgtcgg |
| OsRFC5 Δ1-237 | | gcTCTAGAatgcagcaatacccatttacat | | cgGGATCCgccaaatgtagagacaagaaatt |
| OsRFC5 Δ1-300 | | gcTCTAGAatgttactgaagaaattagactc | | cgGGATCCgccaaatgtagagacaagaaatt |
| OsRFC5 Δ235-354 | | gcTCTAGAatgctgtgggtggacaagta | | cgGGATCCggtctcaaaaaacagtatcg |
| OsRFC5 Δ205-354 | | gcTCTAGAatgctgtgggtggacaagta | | cgGGATCCtttcttcccaatgaactcca |
| OsRFC5 Δ155-354 | | gcTCTAGAatgctgtgggtggacaagta | | cgGGATCCcattgtcctacgaagggaatgc |
| OsPCNA Δ1-20 | | gcGTCGACatggagctggtgacggacgc | | cggGGTACCcgacttcatttcctcatctt |
| OsPCNA Δ1-60 | | gcGTCGACatgcgctgcgaccgcaacct | | cggGGTACCcgacttcatttcctcatctt |
| OsPCNA Δ1-120 | | gcGTCGACatgatcgacagcgagcacctc | | cggGGTACCcgacttcatttcctcatctt |
| OsPCNA Δ1-136 | | gcGTCGACatgccctcgtcgcgctgctcctcc | | cggGGTACCcgacttcatttcctcatctt |
| OsPCNA Δ254-263 | | gcGTCGACatgttggagctgaggcttgt | | cggGGTACCtggcgccagataaaatctaa |
| OsPCNA Δ244-263 | | gcGTCGACatgttggagctgaggcttgt | | cggGGTACCctctgcaatcttatactcaa |
| OsPCNA Δ224-263 | | gcGTCGACatgttggagctgaggcttgt | | cggGGTACCttcagagagcgggcttgcc |
| OsPCNA Δ114-263 | | gcGTCGACatgttggagctgaggcttgt | | cggGGTACCatccgcaatcttatcttggt |
| OsPCNA Δ64-263 | | gcGTCGACatgttggagctgaggcttgt | | cggGGTACCgtcgcagcggtagtgctcga |
| AtRFC2 Δ1-224 | | agaGTCGACatggtcaaccaagaaaacgt | | cgcGGTACCaggtgctttcgctgtttcac |
| AtRFC2 Δ314-333 | | agaGTCGACatggcgtcttcttcatcaac | | cgcGGTACCataagatccaactccatcac |
| AtRFC2 Δ294-333 | | agaGTCGACatggcgtcttcttcatcaac | | cgcGGTACCaagtttcagatactcagcca |
| AtRFC3 Δ1-247 | | agaGTCGACatgaaggaaattacagagga | | cgcGGTACCctttgctgcaccaacaatga |
| AtRFC3 Δ350-369 | | agaGTCGACatgactgagctaacgtcggc | | cgcGGTACCctgcagtttgtcgttgcaac |
| AtRFC4 Δ1-213 | | agaGTCGACatcacatatctgcagagtgc | | cgcGGTACCgaagtcttgagccatttctg |
| AtRFC4 Δ320-339 | | agaGTCGACatggcgccagttcttcagag | | cgcGGTACCaagctgcaagtactcatccg |
| AtRFC4 Δ300-339 | | agaGTCGACatggcgccagttcttcagag | | cgcGGTACCgatcttagccttttgcatgt |
| AtRFC5 Δ1-239 | | cgcTCTAGAatgtatccgttcacaggtaacc | | cgcGGATCCcccaaacgttgaaatgagga |
| AtRFC5 Δ335-354 | | cgcTCTAGAatgttgtgggtcgacaagta | | cgcGGATCCtatgtgaaatatggctttct |
| AtPCNA1 Δ1-20 | | agaGTCGACatggatctggtgaacgatgc | | cgcGGTACCgggattagtgtcttcttctt |
| AtPCNA1 Δ1-120 | | agaGTCGACatgatagacagtgaacatct | | cgcGGTACCgggattagtgtcttcttctt |
| AtPCNA1 Δ1-136 | | agaGTCGACatggtgaggatgccttccaa | | cgcGGTACCgggattagtgtcttcttctt |
| AtPCNA1 Δ244-263 | | agaGTCGACatgttggagctacgtcttgt | | cgcGGTACCctcagcaaccttatactcca |
| AtPCNA1 Δ224-263 | | agaGTCGACatgttggagctacgtcttgt | | cgcGGTACCgtctgacaatggagttgcc |
| AtPCNA1 Δ144-263 | | agaGTCGACatgttggagctacgtcttgt | | cgcGGTACCaaactcattggaaggcatcc |
| AtPCNA2 Δ1-20 | | agaGGATCCatggatctggtgaacgatgc | | cgcGGTACCttctggtttggtgtcttctt |
| AtPCNA2 Δ1-120 | | agaGGATCCatgatcgacagtgagcattt | | cgcGGTACCttctggtttggtgtcttctt |
| AtPCNA2 Δ1-136 | | agaGGATCCatggtgagaatgccttctgg | | cgcGGTACCttctggtttggtgtcttctt |
| AtPCNA2 Δ245-264 | | agaGGATCCatgttggagcttcgtttagt | | cgcGGTACCcatctcagccaccttgtact |
| AtPCNA2 Δ225-264 | | agaGGATCCatgttggagcttcgtttagt | | cgcGGTACCagcctttgtgaaggagttca |
| AtPCNA2 Δ145-264 | | agaGGATCCatgttggagcttcgtttagt | | cgcGGTACCggaaaattcaccagaaggca |
